# Metal Packaging: From Monolithic Containers to Hybrid Architectures

**DOI:** 10.3390/ma19061177

**Published:** 2026-03-17

**Authors:** Leonardo Pagnotta

**Affiliations:** Department of Mechanical, Energy and Management Engineering, University of Calabria, Arcavacata, 87036 Rende, CS, Italy; leonardo.pagnotta@unical.it

**Keywords:** metallic packaging, aluminum cans, tinplate, food-contact materials, corrosion and migration, surface coatings, regulatory compliance, circular economy, recyclability, sustainability

## Abstract

Metal packaging materials remain fundamental across food, beverage, pharmaceutical, cosmetic, and technical sectors owing to their combination of mechanical robustness, total light and gas barrier performance, thermal resistance, and established recyclability. Aluminum alloys, tinplate, tin-free steel (TFS/ECCS), stainless steels, metal–matrix composites (MMCs), and metal–polymer or metal–paper laminates define distinct metal-based packaging architectures whose metallurgical and interfacial design governs forming behaviour, corrosion and migration pathways, coating integrity, and mechanical reliability. In this review, these architectures are examined from a materials- and systems-oriented perspective, linking composition, microstructure, processing routes, and surface engineering to functional performance across rigid, semi-rigid, and flexible formats. The analysis also considers the ongoing transition from bisphenol A (BPA)-based epoxy linings to BPA-free and hybrid coating chemistries, the use of nano-structured metallic and metal-oxide surfaces, and the role of composite laminates in which thin metallic foils are combined with polymeric or paper-based structural layers. These material and architectural aspects are discussed together with safety, regulatory, and circularity considerations that increasingly influence the design and selection of metal-based packaging. Ion migration, coating degradation, and corrosion under realistic storage environments are considered in relation to EU, FDA, ISO, and sector-specific requirements, while attention is also paid to the contrast between well-established closed-loop recycling infrastructures for aluminum and steel and the more complex end-of-life management of coated metals and multilayer laminates. The review provides a unified framework connecting materials selection, metallurgical design, processing, performance, regulatory compliance, and sustainability in metal-based packaging systems. Applications spanning consumer goods, pharmaceuticals, cosmetics, and advanced electronics are integrated to support an overall understanding of how metallic and hybrid metal-based architectures underpin functional reliability and life-cycle sustainability.

## 1. Introduction

Metal-based packaging systems have played a central role in the protection, preservation, and distribution of goods for more than a century, supporting food, beverage, pharmaceutical, cosmetic, and technical applications through their unique combination of complete barrier performance, mechanical robustness, and thermal stability [1,2,3]. Metals exhibit an intrinsically dense and continuous atomic structure, providing an essentially impermeable barrier to gases and moisture and complete opacity to light [1]. In packaging, however, this intrinsic barrier must be translated into system-level barrier integrity, since containers and laminates typically include seams, closures, and coated interfaces [4,5]. The mechanical robustness, thermal stability, and process compatibility of metals enable this barrier continuity to be maintained during high-speed forming and under thermal processing conditions [6]. This combination of characteristics has historically positioned metals as reference solutions for long-term storage, sterilization, and safety-critical packaging applications.

Early metal packaging technologies relied on essentially monolithic metal containers—such as steel cans and tinplate formats—whose performance depended primarily on the bulk properties of the metal and relatively simple surface treatments [2,3]. Over time, however, increasing demands related to product safety, functional performance, material efficiency, and regulatory compliance have driven a progressive evolution toward system-level packaging architectures [2,7]. In contemporary applications, metals rarely operate as isolated materials; instead, they function as integral components of hybrid systems incorporating coatings, polymeric layers, paper-based supports, and functional surface modifications. Within these architectures, metallic substrates continue to provide the primary barrier and mechanical backbone, while complementary layers govern sealing reliability, chemical compatibility, and the integration of additional functions required by specific application domains [4].

In this evolving landscape, aluminum alloys, tinplate, tin-free steel (TFS/ECCS), stainless steels, and advanced metal–matrix composites (MMCs) constitute the principal metal-based packaging architectures employed across rigid, semi-rigid, and flexible formats. Their functional behaviour is governed not only by alloy composition but also by crystallographic texture, microstructural evolution, surface chemistry, and processing history. For example, texture anisotropy and strain-hardening behaviour control the formability and earing response of aluminum can-body stock [6], while the development of Fe–Sn intermetallic layers and passivation treatments governs the corrosion resistance and durability of tinplate systems [4,5,8]. In TFS materials, the stability and adhesion of chromium-based duplex layers play a key role in defining coating compatibility and long-term performance, particularly under the combined effects of corrosion and processing-induced stresses [9]. Stainless steels and metal–matrix composites (MMCs) further extend the performance envelope of metal-based packaging toward reusable, high-temperature, and technical applications, including stainless-steel containers for food and pharmaceutical products and electronic housings, where chemical inertness, durability, and reliability are critical [10,11,12,13].

Beyond structural and barrier performance, migration and safety considerations represent a critical regulatory dimension of metal-based packaging [14]. Aluminum intake and metal ion release continue to be monitored internationally, with significant variability in exposure limits and assessment methodologies across regulatory frameworks [15]. Organic acids, elevated temperatures, and coating discontinuities have been shown to accelerate localized corrosion and migration phenomena even in coated systems, highlighting the central role of surface engineering in food and beverage applications [4,16,17]. Electrochemical studies on aluminum beverage cans have also shown that soft drinks with lower pH and higher conductivity, such as cola-based beverages, promote significantly higher corrosion activity compared with less acidic drinks [18]. Further studies have shown that the corrosion behaviour of aluminum beverage cans is also influenced by catalytic ions naturally present in beverages, such as chloride and iron, as well as by alloy composition and coating properties [19].

Parallel concerns apply to coating-related migrants, including bisphenols, phthalates, and non-intentionally added substances (NIAS), whose release depends on coating formulation, storage conditions, and multilayer architecture [20,21]. In response to these concerns, recent research has investigated alternative polymer systems such as polyester-based coatings designed to replace conventional BPA-containing epoxy linings in metal food packaging [22].

At the same time, sustainability and circularity increasingly influence material selection and design strategies for metal packaging. Aluminum and steel benefit from well-established closed-loop recycling infrastructures; however, the growing use of organic coatings and multilayer laminates introduces additional challenges for end-of-life management and material recovery. Studies on complex aluminum-based waste streams demonstrate that metal recovery remains technically feasible, reinforcing the strategic role of metals within circular-economy frameworks [16].

Against this background, the literature on metal packaging remains fragmented, often addressing materials, coatings, regulatory aspects, or sustainability in isolation. The present review addresses this gap by adopting a materials- and systems-oriented perspective, explicitly linking metallurgical design, processing routes, surface engineering, functional performance, regulatory compliance, and circularity within a unified framework. Rather than treating metals as standalone substrates, this work analyzes metal-based packaging as integrated architectures whose performance emerges from the interaction between bulk materials, surfaces, and complementary layers.

**Figure 1:**
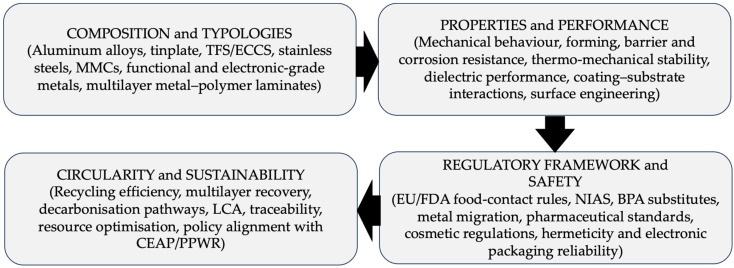
Conceptual roadmap of the review, organized in four domains: composition and typologies, properties and performance, regulatory framework and safety, and circularity and sustainability for metallic packaging systems.

The review is structured according to the conceptual roadmap illustrated in Figure 1, which organizes the discussion into four interconnected domains: (i) composition and material typologies, (ii) properties and functional performance, (iii) regulatory framework and safety considerations, and (iv) circularity and sustainability. This structure supports a coherent analysis of both established and emerging metal-based packaging systems across food, pharmaceutical, cosmetic, and advanced technical applications.

### 1.1. Historical and Technological Evolution of Metal Packaging

Before the emergence of true metallic packaging, metals were extensively used in proto-packaging roles. Across Bronze Age, classical and medieval contexts, containers made of bronze, copper or pewter served as durable vessels for liquids, spices, ointments and ceremonial substances, while metal caskets and boxes protected jewellery, documents and other valuables during transport. Although these artifacts were not “packaging” in the contemporary industrial sense, they already fulfilled the essential functions of containment, mechanical protection and controlled storage that would later be formalized in industrial metal packaging technologies.

The technological evolution of metallic packaging, in the strict sense, reflects a long progression from early tin-coated iron sheets to today’s advanced multilayer and high-performance systems. Historical surveys indicate that tin plating was practised in Central Europe from the late Middle Ages, with tin-coated iron sheets reported in Bohemia and Bavaria by the 14th century, although production remained artisanal and geographically restricted for several centuries [23,24]. Early tins were primarily used for snuff, ointments and dry commodities rather than food, due to limitations in soldering quality, hygiene and concerns about metal toxicity [23].

A decisive transition occurred in the early 19th century with the emergence of heat-processed foods in hermetically sealed containers. Appert’s thermal preservation process, introduced in France in 1809 to address military supply needs, led directly to the adoption of metal vessels for food sterilization; shortly thereafter, Peter Durand patented the first cylindrical tinplate can in 1810, marking the beginning of industrial canning [23,25]. By 1812, the first commercial canning factory had been established in London, signalling a shift from artisanal methods to early mechanized production [26]. Throughout the 19th century, improvements in soldering, the introduction of interior enamels, and later, the development of the double seam in the 1880s increased sealing reliability and reduced contamination risks [23,25].

Metal packaging diversified rapidly during the late 19th and early 20th centuries. Printed tins for confectionery, bakery products and toiletries enabled the integration of lithography with rigid metal formats, supporting emerging branding and marketing strategies [23,24]. In the United States, the canning industry expanded dramatically: by the early 20th century, annual production reached tens of millions of base-boxes of tinplate, with a large fraction dedicated to food processing [27]. Industrial adoption accelerated with continuous body-making, improved welding processes and better control of tin coating thickness, which enhanced forming behaviour and corrosion performance [25]. Opening systems also evolved significantly: from key-wind tear strips in the 1860s to dedicated can openers in the 1870s, and eventually to ring-pulls and stay-tabs introduced in the 1960s–1970s, which transformed beverage can usability [23,25].

A major technological shift occurred with the industrial availability of aluminum. Although aluminum extraction was demonstrated in the 19th century, commercial aluminum foil entered the market only around 1910; aluminum foil containers appeared in the early 1950s, and the first aluminum beverage cans were introduced by the end of that decade [23,24]. Aluminum’s low density, excellent formability and compatibility with deep-drawing and ironing processes enabled the development of lightweight two-piece cans that rapidly reshaped the beverage sector [7,25]. In parallel, aluminum foil became a key material for pharmaceutical blisters and multilayer laminates, offering exceptional moisture and gas barrier properties when combined with polymer heat-seal layers [7,25].

From the late 20th century onward, innovation in metal packaging shifted toward surface engineering, alloy optimization and multilayer structures. Electrolytic tinplate benefited from refined passivation and improved control of Fe–Sn intermetallic growth; tin-free steel (TFS/ECCS) provided chromium-based alternatives with superior coating adhesion and formability; and internal linings evolved from BPA-epoxy systems to polyester, acrylic and hybrid chemistries aimed at reducing migration while maintaining corrosion protection [2,23]. In aluminum packaging, the differentiation of alloys for can body, end and tab applications improved drawability, earing behaviour and mechanical stability. Metal–polymer laminates further broadened the use of thin-gauge foils in both flexible and semi-rigid formats [7,25]. This technical evolution progressively transformed metal packaging from predominantly monolithic containers into system-level architectures, in which metallic substrates, coatings, and complementary materials operate as integrated functional assemblies.

Beyond the food and beverage sectors, metals became integral to specialized and high-performance applications. Beer canning, introduced in the 1930s, required coatings resistant to acidity, carbonation and pasteurization stresses, driving advances in internal linings and external varnishes [28]. In pharmaceuticals, aluminum laminates established themselves as the dominant material for moisture-sensitive formulations. More recently, metallic housings have become essential in MEMS, sensors and microelectronic devices, where hermeticity, residual-gas control and thermo-mechanical compatibility are critical [13,29].

From an environmental standpoint, metals were among the first packaging materials to be recovered at an industrial scale, aided by their magnetic or density-based separability. Steel and aluminum maintain some of the highest recycling rates globally, and emerging delamination technologies now enable the recovery of aluminum from complex multilayer structures such as pharmaceutical blisters, reinforcing the strategic position of metals in circular-economy policies [7,25].

**Figure 2:**
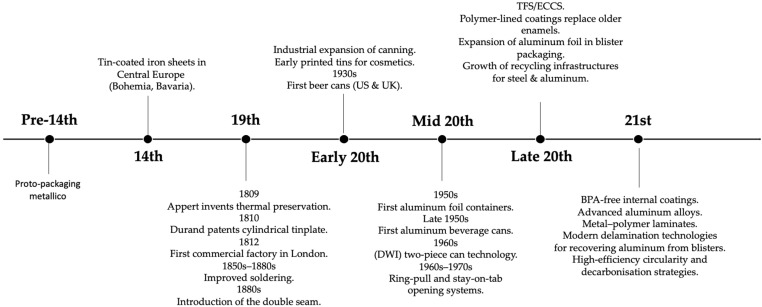
Historical evolution of metal packaging technologies. Timeline of key milestones from early tin-coated iron sheets to modern alloys, coatings, laminates, and recycling routes.

Figure 2 summarizes this historical trajectory, highlighting the transition from early tin-coated iron sheets to today’s metal-based packaging architectures, where advanced alloys, coatings, laminates and recycling infrastructures jointly define the contemporary technological landscape.

### 1.2. Methodological Note and Scope

This review follows PRISMA 2020 principles adapted to a mixed qualitative–quantitative synthesis of metallic packaging systems, aiming at a traceable materials- and systems-level interpretation combining metallurgy, surface engineering, safety, circularity, and regulation. The literature search (updated to 2025) was conducted using Scopus, Web of Science, ScienceDirect, and Google Scholar, complemented by publisher platforms and institutional/regulatory repositories, using keyword strings covering material families, processing, corrosion/migration, coatings, and end-of-life routes.

**Figure 3:**
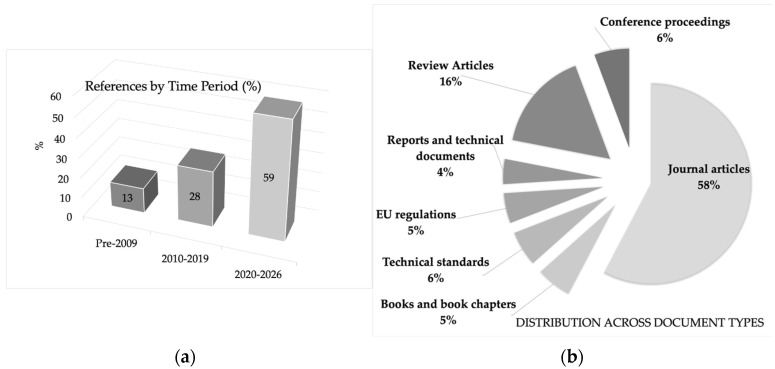
Overview of the literature dataset used in this review. (**a**) Temporal distribution of anlyzed scientific references. (**b**) Distribution of source types (peer-reviewed articles/reviews, conference papers, books/chapters, and normative documents).

The analyzed dataset includes 110 dated scientific references (1918–2026) and several normative sources (technical standards, EU regulations and guidance, and reports/technical documents). As shown in Figure 3a, the scientific references are intentionally weighted toward recent publications to capture current technological, regulatory, and circular-economy developments; this temporal distribution reflects a selection strategy rather than a bibliometric trend of scientific growth. The document-type composition reported in Figure 3b mirrors the categories used in this review, distinguishing peer-reviewed research and review articles from conference contributions, books/chapters, and normative sources that define standardized test methods and compliance requirements.

## 2. Material Families and Typologies

Metal packaging materials constitute a heterogeneous class of engineered systems whose performance arises from the interplay between alloy composition, microstructure, surface chemistry, and forming processes.

Three conventional metallurgical families dominate industrial metal packaging: aluminum alloys, steel-based systems (including tinplate and tin-free steel/ECCS), and stainless steels [12,25]. Each is defined not only by bulk composition but by characteristic surface architectures and processing routes, which ultimately determine forming behaviour, corrosion pathways, coating adhesion, and suitability for specific packaging functions [7,25]. Alongside these systems, advanced metal-based composite architectures extend the performance domain of metallic packaging. These include both metal–matrix composites (MMCs), such as Al–SiC and Al–diamond used in hermetic and electronic housings [13,30], and metal–polymer or metal–paper laminates, where aluminum or steel foils are integrated with organic layers to provide flexible or semi-rigid, high-barrier solutions widely employed in food, beverage, and pharmaceutical applications [7,25].

Beyond bulk substrates, several metallic species are employed predominantly as functional surface layers rather than structural materials. Tin, chromium, nickel, copper, silver, and gold appear in electrolytic coatings, passivation treatments, metallizations, antimicrobial layers, or sensing surfaces, particularly in food-contact, pharmaceutical, and electronic packaging [31,32,33].

Although these layers do not significantly contribute to load-bearing capacity, they critically influence corrosion resistance, interfacial adhesion, migration behaviour, functional performance, and recyclability, and are therefore discussed in the context of surface engineering, coatings, and smart or hermetic packaging systems [1,32].

The functional behaviour of metallic packaging is governed as much by surface architecture as by alloy type. Aluminum relies on its native Al_2_O_3_ passive film, which controls corrosion resistance and provides an adhesion platform for lacquers and laminates. Tinplate performance derives from its multilayer structure consisting of a steel substrate, Fe–Sn intermetallic layer, metallic tin coating, and passivation treatments [8]. Tin-free steel utilizes a Cr/Cr-oxide duplex system, providing chemical stability and excellent lacquer adhesion [1], whereas stainless steels depend on the stability of a self-healing Cr_2_O_3_ passive film to ensure long-term inertness [20]. In laminated architectures, barrier and durability depend on both metallic and polymeric layers and on interfacial compatibility across the multilayer system [7,25].

Processing routes exert a decisive influence on performance. Cold rolling, annealing, tempering, and textural control define the mechanical and forming behaviour of aluminum and steel sheets, while deep drawing, ironing, welding, and double seaming introduce severe deformation histories unique to packaging. Microstructural gradients, residual stresses, and surface conditioning govern formability, barrier integrity, and sealing reliability [5,6]. In MMCs, manufacturing routes determine reinforcement distribution and interfacial chemistry, whereas in laminated systems, bonding processes, heat-sealing, and delamination behaviour control functional stability and end-of-life recovery efficiency [7,25,34,35].

Metal packaging, therefore, encompasses a broad landscape of monolithic and composite metallic architectures whose functional properties arise from their metallurgical design and surface engineering.

**Figure 4:**
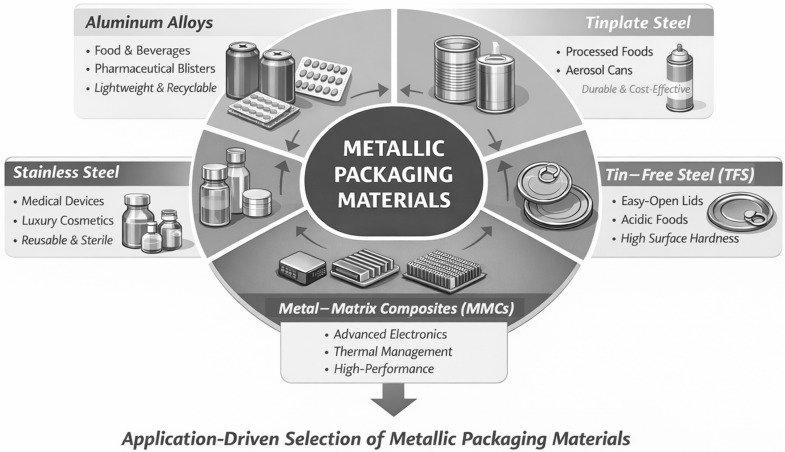
Metallic packaging material families and typical application domains. Qualitative, application-oriented overview of the main material families considered in this review.

Figure 4 provides a conceptual overview of the main metallic packaging material families and their typical application domains, offering an orientation framework for the detailed classification and analysis presented in the following figures and subsections.

**Figure 5:**
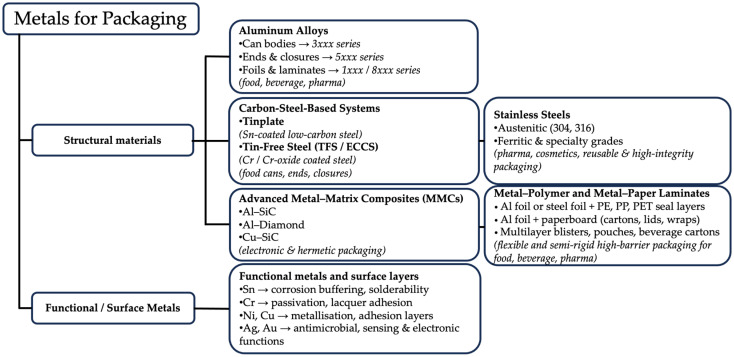
Classification of metallic materials used in packaging. Structural families (Al alloys, tinplate, TFS/ECCS, stainless steels, and MMCs) and functional metallic surface components (e.g., tin, chromium, and nickel).

Figure 5 classifies the material families considered in this review. The following subsections are organized by material family and apply a consistent analytical framework addressing composition and architecture, microstructure and processing routes, intrinsic barrier and mechanical properties, surface chemistry and coating interactions, functional and sealing performance, and packaging applications (Figure 4).

**Figure 6:**
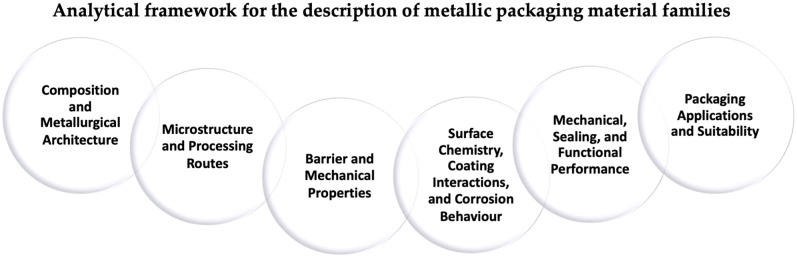
Analytical framework used for material-family sections. Common sequence adopted for all families: composition/architecture, processing/microstructure, properties, surface/coatings/corrosion, functional performance, and applications.

**Figure 7:**
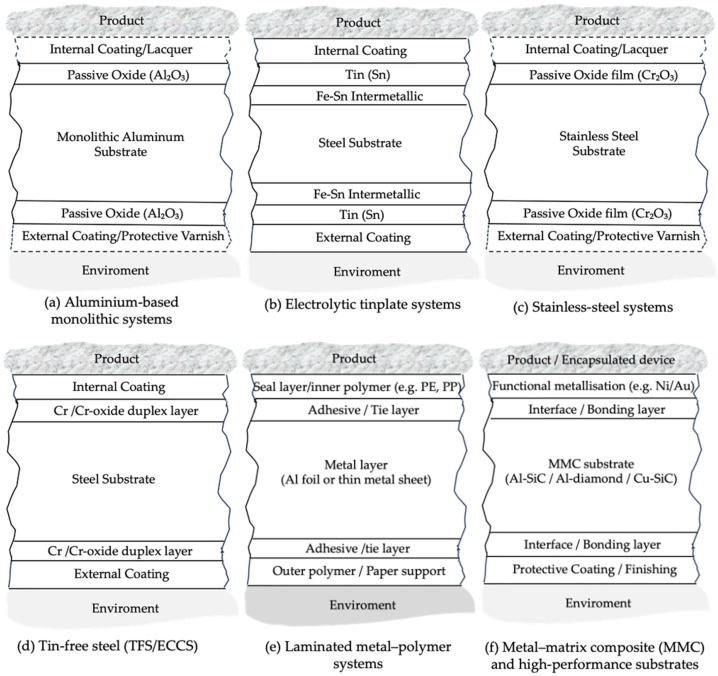
Structural archetypes of metal-based packaging systems. (**a**) Aluminum-based monolithic systems: metallic substrate with native oxide film and internal/external protective coatings. (**b**) Electrolytic tinplate systems: low-carbon steel substrate coated on both sides with Fe–Sn intermetallic layers, metallic Sn and polymeric lacquers. (**c**) Stainless-steel systems: corrosion-resistant steel with self-healing Cr_2_O_3_ passive films and optional protective coatings. (**d**) Tin-free steel (TFS/ECCS): steel substrate with duplex Cr/Cr-oxide passivation layers and organic coatings ensuring corrosion protection and lacquer adhesion. (**e**) Laminated metal–polymer systems: multilayer architectures in which aluminum foil acts as a high-barrier layer embedded between polymer seal layers, adhesives and external polymer/paper supports. (**f**) Metal–matrix composite (MMC) and high-performance substrates: engineered metallic materials (e.g., Al–SiC, Al–diamond, Cu–SiC) providing tailored thermo-mechanical stability, metallization compatibility and environmental protection for hermetic and advanced technical packaging applications.

For all material families discussed in Section 2, the analysis follows the same structured sequence, schematically illustrated in Figure 6. In addition, the main packaging architectures associated with the metallic systems discussed in Section 2 are schematically compared in Figure 7.

### 2.1. Aluminum Alloys

Aluminum alloys represent one of the most versatile material families used in packaging, including rigid containers, beverage cans, closures, foils, and pharmaceutical laminates. Their widespread adoption is driven by the combination of low density, high formability, intrinsic corrosion resistance and compatibility with high-throughput forming operations. Packaging applications rely predominantly on non-heat-treatable aluminum alloys, whose mechanical behaviour is governed by solid-solution strengthening and strain hardening rather than precipitation mechanisms. In practice, alloys of the 1xxx, 3xxx, 5xxx and 8xxx series dominate the sector (AA designation: the first digit identifies the main alloying family; 1xxx ≈ commercially pure Al, 3xxx = Al–Mn, 5xxx = Al–Mg, 8xxx = other foil-oriented systems). 1xxx and 8xxx grades are used mainly for foil products [1], whereas 3xxx and 5xxx alloys provide the microstructural stability and deformation behaviour required for deep drawing, ironing and repeated bending without fracture [2,6,25].

#### 2.1.1. Composition and Metallurgical Architecture

The non-heat-treatable 1xxx, 3xxx, 5xxx and 8xxx aluminum families are tailored to satisfy the competing requirements of deep drawability, resistance to local thinning, sealing reliability and high-barrier performance [6].

Alloys of the 1xxx series (commercially pure Al) are used where maximum ductility, cleanliness and surface uniformity are required, particularly in thin-gauge foils and lidding materials, and as a substrate for laminated barrier structures [1]. Alloys of the 3xxx series (notably AA3004 and AA3104) combine manganese in solid solution with finely dispersed Mn-bearing particles, stabilizing recrystallization textures and enabling can-body wall thicknesses below 0.1 mm while still supporting hoop stresses generated by carbonation [6]. 5xxx alloys, in particular AA5182, provide higher yield strength and work-hardening capacity, allowing complex scoring, rivet-forming and opening operations in can ends without premature cracking [6]. 8xxx foil alloys contain finely dispersed Fe–Si intermetallic particles that enhance tear resistance and limit pinhole formation, which is critical for pharmaceutical blisters and retortable laminates [1,2]. A defining feature of aluminum packaging materials is the spontaneous formation of a nanometric Al_2_O_3_ passive film, which is central not only to corrosion resistance but also to adhesion of internal coatings and to seal development in multilayer laminates. As a result, surface chemistry is an integral component of the overall metallurgical architecture rather than a secondary surface attribute [1].

#### 2.1.2. Microstructure and Processing Routes

The microstructural state of packaging-grade aluminum is inseparable from its processing history. Cold rolling and intermediate annealing generate well-defined recrystallization textures—predominantly Cube, with variable contributions from Goss, Brass and S components (i.e., characteristic crystallographic orientations that control planar anisotropy and drawability)—that directly determine the material’s ability to undergo deep drawing without strain localization. Texture gradients across the sheet thickness, extensively documented in can-body stock, govern ear formation, wall-thickness uniformity and the onset of localized thinning during ironing [6].

During drawn-and-ironed (DWI) operations, aluminum experiences very large plastic strains under biaxial and plane-strain conditions. The resulting microstructure must retain sufficient work-hardening capacity to resist buckling under internal pressure while maintaining enough ductility to prevent splitting at the base and shoulder regions. For ends and closures, higher-strength 5xxx alloys must accommodate rivet forming, scoring and controlled tearing during opening, operations in which local anisotropy and residual stresses strongly influence sealing reliability.

In foil products, extreme thickness reductions amplify the role of inclusions, Fe–Si dispersoids and grain morphology. These features regulate pinhole density, tear propagation pathways and resistance to flex cracking in pharmaceutical and retort laminates, making microstructural cleanliness and control essential for functional performance [1,2].

#### 2.1.3. Barrier and Mechanical Properties

Aluminum alloys combine excellent barrier performance with a favourable strength-to-weight ratio. Their dense metallic lattice ensures near-zero permeability to gases, light and moisture, underpinning their widespread use in both rigid containers and flexible multilayer systems. In foil form (typically 6–20 μm), aluminum provides oxygen transmission rates below ~0.02 cc·m^−2^·24 h^−1^, enabling extended shelf life for oxygen-sensitive foods and pharmaceutical products [1,2].

In rigid packaging, the mechanical behaviour of 3xxx and 5xxx alloys supports thin-wall deformation without fracture. Typical tensile strengths range from approximately 130 to 300 MPa, while the Young’s modulus (~70 GPa) allows stable deep drawing and ironing at industrial speeds. These properties ensure dimensional integrity under internal pressure and during thermal treatments such as pasteurization and retort.

Corrosion resistance derives from the stability of the alumina film; however, acidic or chloride-rich environments may induce localized pitting where coating defects expose bare metal. Recent studies demonstrate accelerated corrosion in beverages containing organic acids, such as sour beers, where lactic and acetic acids promote crevice attack even in coated containers [17]. Earlier investigations on carbonated soft drinks reported similar trends, linking localized corrosion to organic acids, dissolved CO_2_ and chloride ions acting at coating discontinuities [36,37].

From a food-contact perspective, dietary exposure studies indicate that aluminum migration from properly coated containers generally remains limited under standard conditions, while localized corrosion at coating defects represents the dominant risk pathway in acidic and fermented products [15,38].

Barrier performance, therefore, depends critically on the quality and continuity of internal coatings.

#### 2.1.4. Surface Chemistry, Coating Interactions, and Corrosion Behaviour

Although aluminum benefits from a protective passive film, its stability can be challenged by chloride-rich food systems, acidic media and sulfur-containing volatiles generated during thermal processing. Under retort conditions (approximately 115–130 °C), partial hydration of the oxide to boehmite modifies surface energy and may weaken adhesion of internal lacquers, affecting both corrosion resistance and sealing performance.

Failure analyses show that localized defects in organic coatings act as initiation sites for underfilm corrosion, which can propagate laterally and reduce buckle strength or compromise the tightness of ends subjected to cyclic pressure. Penetration of sulfur-containing volatiles along micro-defects has been identified as a dominant mechanism of pitting in canned foods [4].

In foil-based laminates, coating interactions define seal strength, flex durability and resistance to delamination. The oxide layer, together with rolling-induced surface roughness, establishes mechanical interlocking and chemical bonding with heat-seal polymers. Any modification of the oxide—through mechanical abrasion, hydration or thermal cycling—can therefore influence functional behaviour, including seal integrity during retort and peelability in pharmaceutical blisters. Mechanical modelling and industrial case studies confirm that barrier failure in such laminates is governed not by aluminum permeability but by foil cracking, pinhole evolution and interfacial debonding under bending and thermal cycling [39].

#### 2.1.5. Mechanical, Sealing, and Functional Performance

The mechanical requirements of aluminum packaging are governed not by bulk strength alone but by the ability to sustain thin-wall geometries under combined internal pressure, axial loads, bending, thermal gradients and cyclic deformation.

For can bodies, the allowable reduction in wall thickness is constrained by hoop strength, which controls resistance to carbonation pressures (typically 0.6–0.9 MPa), buckle pressure defining catastrophic wall instability, axial load resistance relevant to stacking and transport, and panel stability, which is highly sensitive to small variations in thinning during ironing. The mechanical envelope defined by yield strength, strain-hardening exponent and elongation sets the limits for safe operation: higher yield strength improves buckle resistance but reduces formability and increases the risk of localized splits, an intrinsic trade-off in can-body design.

Ends manufactured from AA5182 must retain controlled toughness along score lines to ensure predictable opening behaviour while withstanding transient pressure spikes. Local mechanical response in the rivet region directly influences seaming integrity and leak tightness under thermal cycling and transport loads.

Foil-based structures exhibit a distinct set of functional requirements, including tear resistance controlled by dispersoid populations and grain morphology, pinhole density limiting barrier efficiency, resistance to flex cracking during handling, and seal strength and creep stability during sterilization. Mechanical fatigue under cyclic bending and thermal loading can initiate microcracks that propagate through work-hardened grains, reducing barrier integrity even in the absence of corrosion.

Aluminum’s high thermal conductivity facilitates rapid heating and cooling during retort, reducing thermal gradients and limiting thermally induced stresses at seams and closures. However, repeated thermal cycles can modify residual stress states and local yield behaviour, influencing long-term sealing reliability.

#### 2.1.6. Packaging Applications and Suitability

Aluminum alloys are suitable for a wide range of packaging functions due to their combination of low density, excellent formability and intrinsic barrier performance. Drawn-and-ironed 3xxx alloys dominate beverage cans and thin-walled food containers, where their strain-hardening behaviour supports high internal pressures with minimal wall thickness. Foil-based 8xxx alloys underpin pharmaceutical blister packs, retortable multilayers and high-barrier laminates, exploiting aluminum’s impermeability and thermal conductivity.

5xxx alloys, particularly AA5182, remain the standard for can ends and closures, balancing rivet-forming toughness with mechanical strength for reliable seaming. Aluminum’s compatibility with coatings, lacquers and polymer laminates makes it highly versatile, while its ability to withstand thermal processing ensures suitability for processed foods. Its limitations include susceptibility to pitting in chloride-rich or highly acidic media and sensitivity to coating defects during retort; however, these issues are effectively mitigated through appropriate alloy selection, surface treatments and coating design.

To provide a structured and comparative overview of the main aluminum alloy families employed in packaging, Table 1 summarizes their mechanical characteristics, strengthening mechanisms, corrosion behaviour in food environments, coating requirements, and typical industrial applications.

As shown in Table 1, the differentiation among 1xxx, 3xxx, 5xxx, and 8xxx series is governed by the interplay between strengthening mechanism, corrosion sensitivity, and functional requirements, which ultimately determines their allocation across rigid, semi-rigid, and flexible packaging systems.

### 2.2. Tinplate (Tin-Coated Steel)

Tinplate represents one of the most established metallic packaging systems for thermally processed foods, particularly where long shelf life, complete barrier performance, and mechanical robustness under sterilization conditions are required [40]. Unlike aluminum alloys, whose packaging applications rely primarily on lightweighting and passive corrosion resistance, tinplate is based on a deliberately engineered multilayer system in which mechanical strength, electrochemical behaviour and surface functionality are distributed across distinct material components [41].

The tinplate system combines a low-carbon steel substrate, providing stiffness and resistance to deformation, with a thin electrolytic tin coating that governs corrosion behaviour and surface reactivity [40,41]. This architecture enables controlled surface reactivity and corrosion buffering and allows the tuning of surface interactions through differential tin coatings and organic lacquers, depending on product chemistry and processing conditions [5,42]. As a result, tinplate remains widely used in applications involving acidic or complex food matrices and severe thermal cycles, where dimensional stability and sealing reliability are critical [4,5]. This architecture enables controlled surface reactivity and corrosion buffering and allows the tuning of surface interactions through differential tin coatings and organic lacquers, depending on product chemistry and processing conditions [5,42]. As a result, tinplate remains widely used in applications involving acidic or complex food matrices and severe thermal cycles, where dimensional stability and sealing reliability are critical [4,5].

In the following subsections, tinplate is analyzed according to the same structured framework adopted for other metallic packaging families, covering composition and metallurgical architecture, microstructure and processing routes, intrinsic barrier and mechanical properties, surface chemistry and corrosion behaviour, functional performance during processing and storage, and representative packaging applications.

#### 2.2.1. Composition and Layered Metallurgical Architecture

Tinplate is a steel-based packaging material produced from ultra-low-carbon steel sheet coated with a thin layer of electrolytically deposited tin, resulting in a precisely controlled laminated architecture [2]. Although the tin overlay is typically in the micrometre range, the functional structure is defined by a multilayer stack that includes the steel substrate, Fe–Sn intermetallic at the interface, the free Sn overlay, and a final surface passivation [1,8]. The steel substrate provides the mechanical backbone of the system, enabling the stiffness and strength required for forming, flanging and double-seaming, whereas the tin layer governs surface reactivity and establishes the basis for controlled electrochemical behaviour in service [2]. Electrolytic tinning allows the coating weight to be tuned, including the use of differential tinplate configurations in which the internal and external tin weights differ to match food-contact and external-environment requirements [5].

At the steel–tin interface, thermal and/or electrochemical treatments promote the formation of Fe–Sn intermetallic phases—primarily FeSn_2_, with thinner contributions from FeSn—acting as a metallurgical transition layer that supports adhesion and mechanical anchoring of the coating during deformation [5,8]. The outer free-tin overlay contributes to solderability, corrosion buffering and accommodation of microcracking during forming, while passivation treatments (commonly chromate-based in conventional systems) stabilize the surface, mitigate staining phenomena and improve adhesion of subsequent organic coatings [1].

From a packaging perspective, tinplate is supplied either as unlacquered material—where the metallic tin provides the primary surface function—or as lacquered tinplate, where organic coatings establish the dominant barrier against aggressive product chemistries and processing conditions [2,5].

Overall, tinplate is best described as a purpose-engineered multilayer system in which composition and metallurgical architecture are inseparable from surface-engineering choices that determine packaging reliability [1,8].

#### 2.2.2. Microstructure and Processing Routes

Tinplate for packaging applications is produced through a tightly controlled sequence of thermo-mechanical and electrochemical processing steps designed to ensure uniform microstructure, surface quality and coating integrity. The steel substrate is typically manufactured by continuous casting followed by hot rolling, cold rolling and final annealing, either in batch annealing (BA) or continuous annealing (CA) lines. These routes allow precise control of grain size, crystallographic texture and yield strength, which are critical for forming operations such as drawing, flanging and double seaming [1,2,40].

Cold rolling reductions and annealing parameters are selected to balance strength and ductility, producing a fine-grained ferritic microstructure with limited strain ageing susceptibility. This microstructural condition ensures dimensional stability during high-speed can-making processes and limits the development of localized thinning or Lüders band formation during deformation. Skin-pass rolling is commonly applied as a final step to adjust surface roughness and mechanical response, improving formability consistency and surface finish [40].

Following substrate preparation, tin is deposited by electrolytic tinning, a process that enables accurate control of coating weight, distribution and surface morphology. Electrolytic deposition produces a fine-grained tin layer whose thickness can be independently tailored on each side of the strip, allowing the production of differential tinplate grades for optimized internal and external performance. Subsequent reflow treatments may be applied to modify tin morphology, promoting either matte or bright surface finishes depending on downstream requirements [1].

During tinning and reflow, a thin Fe–Sn intermetallic layer develops at the steel–tin interface as a result of solid-state diffusion. Although limited in thickness, this intermetallic zone plays a decisive role in coating adhesion and mechanical integrity during forming and seaming. Excessive intermetallic growth is avoided through strict thermal control, as it may embrittle the interface and degrade coating performance during deformation [5].

Surface passivation constitutes the final step of the tinplate processing route. Conventional chromate-based treatments have historically been employed to stabilize the tin surface, reduce oxidation and staining, and enhance compatibility with subsequent organic coatings. In parallel with regulatory-driven developments, alternative passivation strategies are increasingly implemented to maintain surface stability while reducing environmental impact, without altering the underlying metallurgical architecture of the tinplate system [2,43].

Overall, the microstructure and processing routes of tinplate are inseparable from its packaging performance: mechanical reliability, surface uniformity and coating integrity all stem from the controlled interaction between substrate metallurgy, tin deposition and post-treatment processes, forming a reproducible and industrially robust material platform for food packaging applications.

#### 2.2.3. Barrier and Mechanical Properties

From an intrinsic standpoint, tinplate provides a complete barrier to gases, vapour, light and microorganisms, a feature dictated by the continuity of the steel substrate rather than by the thin metallic coating. The dense, defect-free steel sheet ensures absolute impermeability, making tinplate inherently suitable for long-term preservation of food products and for applications requiring extended shelf life under ambient or thermally processed conditions [1,2].

The intrinsic mechanical behaviour of tinplate is governed by the low-carbon steel base, which defines elastic modulus, yield strength and resistance to plastic deformation. Compared with aluminum alloys used in packaging, tinplate exhibits significantly higher stiffness and rigidity, providing a mechanically stable substrate capable of retaining its geometry during forming, filling and thermal processing. These properties establish the baseline resistance to buckling, panelling and permanent deformation at the material level [2].

The electrolytic tin coating contributes negligibly to load-bearing capacity but plays a complementary mechanical role by accommodating surface strain and microcracking during deformation. The ductility of the tin overlay allows compatible deformation with the steel substrate, reducing the likelihood of coating fracture and local exposure of steel during drawing and seaming operations [1].

In addition to bulk mechanical properties, surface condition and coating uniformity play a decisive role in forming consistency and strain distribution during can-making. The controlled surface roughness imparted by skin-pass rolling, together with the homogeneous tin overlay, promotes stable frictional behaviour during drawing, flanging and seaming operations. This combination supports reproducible deformation without localized thinning or tearing and contributes to the mechanical robustness of tinplate prior to the influence of surface chemistry, corrosion phenomena or functional performance in service [5].

Overall, the mechanical, sealing and functional performance of tinplate packaging systems emerges from the interaction between material properties, surface engineering and joint design.

#### 2.2.4. Surface Chemistry, Coating Interactions, and Corrosion Behaviour

In practical packaging conditions, the effective barrier performance of tinplate depends critically on the continuity and integrity of the surface system formed by the tin coating and any applied organic layers. While the steel substrate provides an absolute bulk barrier, surface chemistry governs the interaction between the container wall, the internal atmosphere, and the packaged product or propellant, thereby controlling corrosion initiation and propagation mechanisms [44].

Tinplate exhibits a characteristic electrochemical behaviour arising from the nobility difference between tin and steel. Under intact conditions, the tin coating primarily contributes to surface stabilization and corrosion buffering, while the overall corrosion performance remains strongly dependent on coating integrity and local defect conditions. However, this protection is effective only within a limited spatial and chemical domain. Localized discontinuities in the tin layer or in organic coatings—such as scratches, pinholes, seam-related defects, or regions of insufficient coating thickness—can disrupt the electrochemical balance and act as preferential sites for localized corrosion, leading to pitting or underfilm attack [5,41].

At the steel–tin interface, the morphology, continuity and thickness of the Fe–Sn intermetallic layer further influence corrosion behaviour by governing local electrochemical coupling and adhesion of the coating system. Detailed surface and interface analyses of commercial tinplate have shown that heterogeneities in intermetallic development and passivation coverage can locally amplify corrosion susceptibility when combined with coating defects or aggressive product chemistries [8,45].

The interaction between surface condition and the internal atmosphere is particularly relevant in sealed metallic packaging systems. Residual oxygen, moisture and reactive species present in the headspace can accelerate corrosion processes at exposed sites, especially when thermal cycles or long storage times promote desorption and redistribution of adsorbed species from internal surfaces. Experimental investigations on metal packages demonstrate that even minor variations in process parameters affecting residual gas content can significantly influence corrosion susceptibility at coating defects, highlighting the coupled role of surface chemistry and internal atmosphere in hermetic tinplate systems [29].

Organic lacquers therefore represent a critical functional component of tinplate across food, beverage and general-line applications. Their primary role is to isolate the metallic surface from direct contact with aggressive media and to homogenize surface reactivity. When properly applied and cured, lacquered tinplate exhibits high resistance to corrosion under a wide range of service conditions. Conversely, localized coating failures can lead to underfilm corrosion and progressive degradation, even when the bulk tin coating remains nominally intact [41,44]. In parallel, bio-based corrosion inhibitors (e.g., plant-derived extracts) are being explored as complementary mitigation strategies for tinplate under aggressive food-relevant media, particularly in brine-like environments [46]. Recent developments in coating technology aim to extend the functional envelope of conventional lacquer systems. Advanced functional coatings are also being explored to enhance corrosion resistance and surface functionality in aggressive food environments [47,48,49]. In parallel, increasing regulatory pressure has driven the development of chromium-free passivation strategies capable of stabilizing tinplate surfaces while reducing environmental and toxicological concerns, without altering the underlying metallurgical architecture of the system [43].

Seam regions represent a particularly sensitive area from a corrosion standpoint. Mechanical deformation during forming and seaming may locally thin or disrupt surface layers, while geometrical complexity promotes retention of moisture or condensable species. Multiple studies have shown that corrosion phenomena often initiate preferentially at these regions, driven by the combined effects of mechanical strain, electrochemical heterogeneity and internal atmosphere rather than by bulk material properties alone [4,5].

Overall, corrosion behaviour in tinplate packaging systems is governed by the interplay between surface chemistry, coating integrity and internal atmosphere, rather than by intrinsic material properties alone. This system-level perspective explains why tinplate performance cannot be assessed solely on the basis of substrate metallurgy or coating weight, and underscores the central role of surface engineering, coating design and process control in ensuring long-term packaging reliability across diverse application domains.

While these surface- and atmosphere-driven mechanisms govern the initiation of corrosion and degradation phenomena, their impact on container integrity ultimately manifests at the functional level through joint reliability, sealing performance and long-term mechanical stability, which are addressed in the following section.

#### 2.2.5. Mechanical, Sealing, and Functional Performance

The functional performance of tinplate packaging systems in service is governed not only by intrinsic material properties but by the integrated response of the steel substrate and joints under mechanical loading, thermal cycling and internal pressure variations. While the high stiffness of the steel base provides a stable structural platform, long-term performance depends on the ability of the container system to preserve hermeticity and dimensional integrity throughout filling, processing, transport and storage.

Sealing performance represents a critical functional requirement across all tinplate packaging formats. Double seams, welded joints, and crimped closures must withstand combined plastic deformation and thermal exposure without loss of tightness. The reliability of these joints is controlled by forming precision, seam geometry and the local behaviour of surface layers, which must accommodate severe deformation without cracking or delamination. Inadequate control of coating thickness, surface cleanliness or seam compression may compromise sealing performance even when bulk material properties remain within specification [5].

Thermal processing imposes additional constraints on functional behaviour. Elevated temperatures and subsequent cooling cycles promote stress relaxation, redistribution of residual stresses and changes in the internal atmosphere of sealed containers. Experimental studies demonstrate that process parameters influencing residual gas content and surface condition can indirectly affect long-term sealing stability and corrosion susceptibility at mechanically stressed regions, particularly in seam areas [29].

Beyond hermeticity, functional performance also encompasses resistance to impact, vibration and fatigue during distribution. The ductile response of the steel substrate, combined with the energy-absorbing capacity of the container geometry, limits damage propagation and supports tolerance to mechanical abuse over extended storage times.

Overall, the mechanical, sealing and functional performance of tinplate packaging systems emerges from the interaction between material properties, surface engineering and joint design. Reliable service behaviour is therefore a system-level outcome rather than a direct consequence of substrate metallurgy alone.

#### 2.2.6. Packaging Applications and Suitability—Tinplate

Tinplate remains a widely adopted material across multiple packaging segments owing to its combination of mechanical robustness, complete barrier performance and proven reliability under demanding processing and service conditions. Its suitability extends beyond conventional food packaging to include beverage containers, aerosol cans (e.g., cosmetic sprays, technical lubricants, foams and insecticides) and so-called general-line packaging, such as containers for paints, adhesives, lubricants and other industrial or household chemical products, where structural integrity, hermetic sealing and long-term stability are critical requirements [44]. In food-packaging applications, the suitability of tinplate is closely linked to its ability to withstand thermal sterilization, vacuum formation and long storage times without loss of dimensional stability or sealing integrity. Experimental investigations confirm that performance degradation rarely originates from the bulk material, but instead from localized surface or coating discontinuities, reinforcing the importance of surface engineering and process control in real packaging environments [41].

Beyond conventional lacquer systems, advanced functional coatings are increasingly investigated to extend the use of tinplate in food-packaging applications involving aggressive product chemistries or extended shelf life. In particular, nanostructured and hybrid coatings based on epoxy matrices reinforced with graphene derivatives, metal oxides or bioactive phases have been shown to enhance corrosion resistance under acidic or complex food conditions and, in some cases, to provide additional antimicrobial functionality. These developments aim to improve chemical stability and hygiene at the food–package interface without modifying the underlying tinplate architecture [47,48,49].

In response to regulatory restrictions on hexavalent chromium, chromium-free passivation strategies are increasingly adopted to preserve surface stability while reducing environmental and toxicological concerns [43].

Tinplate also plays a central role in aerosol and general-line packaging, where containers must withstand sustained internal pressure, mechanical impacts and repeated handling during filling, transport and storage. Aerosol applications typically involve pressurized systems combining propellants—such as compressed gases or hydrocarbon mixtures—with cosmetic, domestic or technical products. In these conditions, the high stiffness and yield strength of the steel substrate provide resistance to deformation and bursting while maintaining dimensional stability and sealing integrity.

General-line packaging refers to rigid metal containers for non-food products such as paints, varnishes, adhesives, oils and industrial chemical formulations. In these applications, surface coatings are selected primarily to ensure chemical compatibility with solvents and active components rather than food-contact safety. The combination of chemical resistance, robust forming behaviour and reliable double-seaming underpins the continued use of tinplate in demanding service environments characterized by long shelf life and mechanical abuse.

When compared with aluminum-based packaging systems, tinplate provides higher mechanical rigidity and greater resistance to permanent deformation, albeit at the expense of higher material density. This trade-off positions tinplate favourably in applications where pressure resistance, dimensional stability and long-term shape retention outweigh lightweighting considerations, such as aerosol containers, general-line packaging and thermally processed food cans. Aluminum-based systems are instead preferred where mass reduction and extreme formability dominate design priorities.

From an application standpoint, tinplate benefits from well-established recycling infrastructures and consistently high recovery rates for steel-based packaging. This aspect is particularly relevant for high-volume applications such as food cans, aerosol containers and general-line packaging, where established collection and recycling streams strongly influence overall environmental performance. Life-cycle assessment studies indicate that the environmental footprint of tinplate packaging is governed primarily by collection efficiency, recycled steel content and coating complexity rather than by the steel substrate itself [50,51]. Consequently, process optimization and surface-engineering strategies offer effective pathways to reduce environmental impact while preserving existing tinplate-based packaging architectures.

Within tinplate systems, only conventional electrolytic tinplate (ETP) and its double-reduced (DR) variant are considered, as these represent the industrially relevant structural configurations for packaging applications, while tin-free steel (TFS/ECCS) is treated separately due to its distinct coating architecture.

To provide a structured comparison of electrolytic tinplate systems used in packaging, Table 2 summarizes their mechanical characteristics, coating architecture, corrosion behaviour, and typical industrial applications.

As shown in Table 2, the performance of tinplate systems is primarily governed by the integrity of the metallic coating and its interaction with the steel substrate, rather than by the intrinsic corrosion resistance of the base material.

### 2.3. Tin-Free Steel (TFS/ECCS)

Tin-free steel (TFS), also known as electrolytic chromium-coated steel (ECCS), represents the principal alternative to tinplate within steel-based packaging systems. Its distinguishing feature is a surface-engineered protection concept, in which adhesion of organic coatings, chemical inertness at the metal–product interface, and surface hardness are prioritized over the sacrificial corrosion behaviour characteristic of metallic tin.

Instead of a tin overlay, TFS employs a thin duplex layer composed of metallic chromium and chromium oxides, deposited electrochemically onto low-carbon steel sheets. Within the system-level architectures considered in this review (Figure 7d), TFS is therefore classified as a coating-dependent steel system, in which corrosion protection and functional reliability are governed primarily by the integrity and adhesion of organic coatings rather than by electrochemical buffering of the metallic layer.

This architecture confers excellent coating adhesion, stable surface chemistry under thermal sterilization, and high resistance to scratching and abrasion. As a result, TFS is particularly suited for packaging components in which surface stability and lacquer performance dominate design requirements, such as lids, ends, closures and drawn components requiring precise scoring [2].

#### 2.3.1. Composition and Metallurgical Architecture

The TFS substrate is the same low-carbon steel used for tinplate; however, the protective function is entirely transferred from a metallic overlay to a surface-engineered chromium-based system. Instead of forming Fe–Sn intermetallic phases, TFS develops a controlled duplex surface architecture composed of:

1. Metallic chromium layer (Cr^0^)—Dense, continuous, and extremely thin, forming a chemically inert and non-reactive interface with the steel substrate.
2. Hydrated chromium oxide/hydroxide layer (CrOOH/Cr_2_O_3_)—Amorphous and chemically stable, providing corrosion resistance and acting as an effective primer for organic coatings.

Here, Cr^0^ denotes metallic chromium deposited electrolytically, distinct from the overlying chromium oxide/hydroxide layer that governs corrosion resistance and lacquer adhesion.

The total chromium-based coating weight is low—typically 30–60 mg m^−2^ per side—yet its influence on surface chemistry and interfacial behaviour is disproportionate to its thickness. The chromium-rich layer system does not dissolve in contact with food; its function is not sacrificial but barrier-forming, establishing stable surface energy and strong affinity for lacquers [1,40].

A key architectural difference from tinplate is that TFS is inherently non-solderable, and all joining operations must rely on mechanical methods. Packaging design has therefore evolved toward purely mechanical sealing strategies, in which seam geometry, substrate strength and coating integrity ensure mechanical tightness without metallurgical bonding.

#### 2.3.2. Microstructure and Processing Routes

Since the steel substrate employed in tin-free steel (TFS/ECCS) is metallurgically identical to that used for tinplate, its intrinsic formability is governed by the same parameters, including ferritic grain size, crystallographic texture developed during cold rolling, and temper grade. These factors control yield strength, elongation, and planar anisotropy, thereby defining drawability, flangeability, and dimensional stability during forming operations [2,52].

The processing route for TFS follows the conventional sequence adopted for packaging-grade steels, comprising continuous casting, hot rolling, cold rolling, and final annealing, with optional skin-pass rolling to adjust surface roughness and mechanical response. This metallurgical route is essentially identical to that used for tinplate prior to surface coating [2].

However, the absence of a ductile metallic tin overlay introduces a fundamental difference in surface–tool interactions during forming. While tinplate benefits from the presence of a soft tin layer that can accommodate localized deformation and facilitate sliding at the tool–metal interface, the chromium-based surface of TFS exhibits higher hardness and lower ductility. As a consequence, forming behaviour becomes more sensitive to lubrication conditions, tool geometry, and surface roughness [53].

Despite these constraints, the chromium/chromium-oxide surface provides superior resistance to scratching and abrasion compared with tin-coated systems. This characteristic is advantageous in high-speed production lines and in operations involving repeated contact with tooling, where tin overlays may suffer from smearing or microcracking. Preservation of surface integrity prior to lacquer application is therefore enhanced in TFS-based systems [53].

During end forming and double seaming, TFS relies entirely on the mechanical response of the steel substrate, without the lubricating or strain-accommodating contribution of metallic tin. Coating adhesion and continuity must therefore be maintained under highly localized plastic deformation. Experimental studies on lacquered TFS surfaces have shown that curing conditions and near-surface chemistry strongly influence adhesion stability and electrochemical behaviour after deformation, highlighting the tight coupling between processing history and surface performance [9,42].

Overall, while the bulk microstructural control of TFS mirrors that of tinplate, its processing behaviour is distinguished by surface-driven constraints. These include greater dependence on coating adhesion, limited tolerance to surface damage, and the need for precise control of lubrication, curing, and seaming conditions to ensure reproducible manufacturing performance.

#### 2.3.3. Barrier and Mechanical Properties

When combined with appropriate organic coatings, tin-free steel (TFS/ECCS) provides barrier performance comparable to that of tinplate, offering effective protection against oxygen, moisture, and light. As for all metallic packaging materials, the barrier function of the steel substrate itself is essentially complete; therefore, barrier capacity in TFS-based systems is governed primarily by coating integrity rather than by the metallic surface layer [52].

The chromium-oxide-based surface of TFS contributes indirectly to barrier preservation by enhancing scratch and abrasion resistance during forming and handling. This characteristic reduces the likelihood of coating damage prior to filling and thermal processing, supporting the maintenance of barrier continuity throughout manufacturing operations [53]. However, in contrast to tinplate, TFS does not provide sacrificial electrochemical protection, and any exposure of the steel substrate due to coating defects can lead to rapid localized corrosion. As a consequence, the organic coating system represents the primary corrosion barrier in TFS packaging architectures [42,54].

From a mechanical standpoint, the load-bearing capacity of TFS is dictated by the steel substrate and is therefore comparable to that of tinplate with similar temper grades. Yield strength, elastic modulus, and elongation define resistance to stacking loads, internal pressure, and deformation during seaming and thermal cycling. The absence of a ductile tin overlay, however, modifies surface strain accommodation, increasing the sensitivity of TFS systems to coating continuity under severe localized deformation [2].

Under retort and sterilization conditions, lacquered TFS exhibits stable mechanical and barrier performance provided that curing conditions are optimized. Experimental studies have shown that curing temperature and time influence near-surface chemistry and adhesion strength, thereby affecting the electrochemical response of deformed coated areas after thermal exposure [42]. These observations highlight the importance of coating performance and curing conditions for long-term barrier stability.

Overall, while the intrinsic barrier properties of TFS-based packages are equivalent to those of tinplate when coatings remain intact, their mechanical and barrier performance is more strongly dependent on coating quality, adhesion, and defect control, reflecting the coating-dependent nature of the TFS protection concept.

#### 2.3.4. Surface Chemistry, Coating Interactions, and Corrosion Behaviour

The surface chemistry of tin-free steel (TFS/ECCS) is defined by its chromium-based duplex layer, which provides a chemically inert and energetically favourable interface for organic coatings. The outer chromium oxide/hydroxide layer exhibits high stability across a wide pH range and promotes strong interfacial bonding with epoxy–phenolic and related lacquer systems, resulting in superior coating adhesion compared with tinplate under both mechanical deformation and thermal exposure [53].

In contrast to tinplate, corrosion behaviour in TFS-based systems is not influenced by sacrificial metal dissolution or galvanic buffering. Chromium-coated steels do not provide electrochemical protection to exposed steel areas, and corrosion resistance therefore depends on the integrity and adhesion of the organic coating. Electrochemical studies on Cr(III)-based coated steels demonstrate that, in the absence of an intact organic overlayer, localized corrosion initiates rapidly at coating defects due to direct exposure of the steel substrate [9,52].

Coating–substrate interactions are consequently central to corrosion control in TFS packaging. The chromium oxide surface forms chemically robust bonds with organic coatings, limiting underfilm corrosion and delamination when coating continuity is preserved. However, the effectiveness of this interface is strongly dependent on processing parameters, particularly coating formulation and curing conditions. Experimental investigations under simulated retort environments show that elevated temperature and prolonged thermal exposure can modify interfacial adhesion and accelerate degradation processes, especially in acidic or chloride-containing media [11].

Under sterilization and retort conditions, properly cured lacquered TFS exhibits stable electrochemical behaviour and low corrosion rates, provided that coating integrity is maintained during forming and seaming. Conversely, sub-optimal curing or mechanical damage introduced during processing can compromise adhesion, creating preferential pathways for electrolyte ingress and localized corrosion. These observations highlight the tight coupling between surface chemistry, coating performance, and processing history in determining long-term corrosion resistance [9].

Overall, corrosion behaviour in TFS packaging systems is governed primarily by coating performance and defect control. The chromium-based surface architecture provides an inert and adhesion-promoting interface, but long-term durability is governed primarily by coating quality, curing optimization, and defect minimization. This fundamental distinction differentiates TFS from tinplate and underpins its use in applications where coating reliability and surface stability are critical.

#### 2.3.5. Mechanical, Sealing, and Functional Performance

The mechanical performance of tin-free steel (TFS/ECCS) is governed primarily by the properties of the low-carbon steel substrate and is therefore comparable to that of tinplate when equivalent temper grades are employed. Yield strength, elastic modulus, and elongation determine resistance to stacking loads, panel deformation, and dimensional stability during thermal processing. The chromium-based surface layer does not contribute to load bearing but influences the distribution of near-surface strain during forming operations [2].

In contrast to tinplate, the absence of a ductile tin overlay reduces the capacity of the surface to accommodate localized plastic deformation. As a consequence, mechanical reliability in TFS-based systems is more strongly coupled to coating continuity, particularly in regions subjected to high strain concentration, such as score lines, flanges, and seam interfaces. This behaviour necessitates careful control of temper grade, surface roughness, and forming parameters to prevent microcracking or loss of adhesion during manufacture [53].

Sealing performance in TFS packaging relies exclusively on mechanical joining methods, as the chromium-based surface is inherently non-solderable. Double seaming and crimping operations must therefore achieve hermeticity through geometric interlocking and controlled plastic deformation of the steel substrate. When coating adhesion is preserved, TFS can deliver seam tightness and leak resistance comparable to tinplate-based systems. However, because corrosion protection is coating-dependent, any coating damage introduced during seaming can directly compromise long-term sealing reliability [52].

From a functional standpoint, TFS offers complete light shielding and magnetic responsiveness, enabling efficient automated handling, sorting, and quality control in high-throughput packaging lines. The chemically inert nature of the chromium oxide surface minimizes metal–product interactions, reducing the risk of flavour alteration, metallic off-notes, or surface staining in sensitive food categories. These attributes are particularly advantageous in applications where surface appearance, coating performance, and dimensional precision are prioritized over sacrificial corrosion protection [9].

Overall, the mechanical and sealing performance of TFS-based packaging systems reflects a surface-controlled functional paradigm. While the steel substrate provides the necessary structural capacity, reliable performance depends on the preservation of coating adhesion and integrity throughout forming, seaming, and service. This balance underpins the preferential use of TFS in packaging components such as ends, lids, and closures, where precise geometry, coating stability, and functional reliability are critical.

#### 2.3.6. Packaging Applications and Suitability—Tin-Free Steel (TFS/ECCS)

Tin-free steel (TFS/ECCS) is primarily employed in packaging applications where coating adhesion, surface stability, and dimensional precision are more critical than sacrificial corrosion protection. Its use is therefore concentrated in components and formats in which the functional performance of the organic coating system governs long-term reliability.

In food packaging, TFS is widely adopted for can ends, lids, and closures, particularly in drawn or scored components. Typical examples include easy-open ends for vegetables, fish, meat products, and ready meals subjected to thermal sterilization. In these applications, the chromium-based surface ensures strong lacquer adhesion and stable score geometry, enabling predictable opening behaviour while maintaining hermetic sealing throughout retort processing. The absence of tin dissolution also avoids sulfide staining and metallic flavour interactions in sulfur-containing or acidic food matrices [52,53].

TFS is also used in two-piece drawn packaging components where precise forming and surface durability are required. Shallow drawn lids, caps, and ends for composite or multi-material containers benefit from the high scratch resistance of the Cr/Cr-oxide surface, which limits coating damage during high-speed forming and handling. In these cases, corrosion protection is fully entrusted to the lacquer system, making coating quality and curing optimization central design parameters [9].

In non-food and general-line packaging, TFS finds application in closures and container components for products such as paints, coatings, lubricants, and household chemicals. Here, chemical compatibility with aggressive formulations and resistance to abrasion during filling and transport are prioritized over food-contact considerations. The strong adhesion between chromium oxides and organic coatings enables the use of specialized lacquer systems tailored to solvent-rich or alkaline environments, while the steel substrate provides the necessary mechanical rigidity.

Aerosol packaging represents a more selective application domain for TFS. While tinplate remains dominant for aerosol bodies due to its ductility and forming tolerance, TFS is used for aerosol ends and components where coating performance, surface hardness, and dimensional accuracy are critical. In these cases, TFS contributes to stable sealing and resistance to handling damage, provided that coating integrity is maintained during forming and crimping operations.

From an application perspective, the suitability of TFS is therefore defined less by the steel substrate itself than by the surface-engineered protection concept that underpins its performance. TFS is preferentially selected where surface stability, lacquer adhesion, and geometric precision dominate design requirements, while tinplate remains favoured in applications demanding sacrificial corrosion buffering or extensive plastic deformation.

Overall, TFS occupies a complementary role within steel packaging systems, serving applications in which functional reliability is governed by surface engineering and coating performance rather than by metallic corrosion protection mechanisms.

To provide a structured technical overview of electrolytic chromium-coated steel (TFS/ECCS) systems used in packaging applications, Table 3 summarizes their coating architecture, mechanical characteristics, corrosion behaviour and typical industrial uses. In contrast to tinplate systems, the performance of TFS is governed primarily by coating adhesion, surface stability and the integrity of organic lacquers rather than by sacrificial electrochemical protection.

As shown in Table 3, the functional reliability of TFS systems is intrinsically linked to coating adhesion and lacquer integrity, rather than to metallic-layer electrochemical buffering. Unlike tinplate, where the tin overlay contributes to corrosion mitigation even in the presence of minor coating defects, TFS relies entirely on the continuity and performance of organic coatings to ensure long-term durability in packaging applications.

### 2.4. Stainless Steels and Specialty Alloys

Stainless steels occupy a specialized and performance-driven role within metallic packaging systems, being selected in applications where chemical inertness, corrosion resistance, hygiene, and long-term mechanical stability are prioritized over lightweighting, cost efficiency, and high-volume formability. Unlike tinplate, tin-free steel (TFS/ECCS), or aluminum alloys, stainless steels are not widely adopted in mass-market disposable food packaging; however, they are essential in reusable containers, pharmaceutical and cosmetic components, closures, valves, pump systems, and packaging architectures requiring repeated sterilization or prolonged service life, where any release of metal ions, coating degradation, or loss of dimensional stability is unacceptable [52,55].

The performance of stainless steels in packaging applications derives from their ability to form stable, self-healing passive films, which suppress metal dissolution and minimize interactions at the product–material interface. This characteristic enables coating-free or minimally coated solutions, as well as resistance to aggressive cleaning and sterilization cycles. Comparative studies on reusable food-contact containers have shown that stainless steel systems can provide superior chemical stability and absence of organic contaminants when compared with coated aluminum or polymer-based alternatives [10,20].

From a compositional standpoint, packaging-relevant stainless steels are predominantly based on austenitic Cr–Ni alloys (e.g., AISI 304 and 316 grades), which combine corrosion resistance, toughness, and sufficient formability for thin-walled components, while ferritic or low-nickel grades may be employed in cost-sensitive or magnetically responsive parts. The corrosion behaviour of these alloys in food-contact environments is governed by passive film stability and is generally characterized by very low metal ion release, remaining within regulatory limits under most service conditions, with recognized limitations in chloride-rich or highly acidic media [48,49,50].

In the context of packaging design, stainless steels should therefore be regarded not as direct substitutes for tinplate or aluminum, but as specialty materials enabling durability-oriented, reusable, and high-reliability packaging solutions, where functional performance and chemical neutrality dominate material selection criteria.

#### 2.4.1. Composition and Metallurgical Architecture

The stainless-steel families relevant to packaging applications are primarily represented by austenitic and ferritic grades, selected according to the required balance between corrosion resistance, formability, and cost. Austenitic stainless steels, such as AISI 304 and 316, are the most widely employed due to their combination of corrosion resistance, toughness, and processability. These alloys typically contain 18–20 wt.% Cr and 8–12 wt.% Ni, with molybdenum additions in AISI 316 further enhancing resistance to localized corrosion in aggressive or chloride-containing environments. Ferritic stainless steels, exemplified by AISI 430, contain 14–17 wt.% Cr without Ni, and are occasionally adopted in cost-sensitive or magnetically responsive components where moderate corrosion resistance is sufficient.

In addition to these standard grades, specialty corrosion-resistant alloys, including Mo- or N-enriched compositions, are used in highly demanding packaging-related components such as pharmaceutical dispensing systems, atomizing pumps, valves, and high-integrity housings, where dimensional stability and resistance to chemical attack are critical.

The defining characteristic common to all stainless steels is the formation of a thin, continuous, and self-healing Cr_2_O_3_-based passive film, typically 1.5–3 nm thick, which forms spontaneously in the presence of oxygen. The chemistry and protectiveness of this passive layer adapt dynamically to environmental conditions such as temperature, humidity, and oxygen availability. As a result, the passive film governs not only corrosion resistance but also metal–product interactions, influencing resistance to staining, sulphide attack, and flavour modification in food-contact applications [56,57,58].

In contrast to tinplate or tin-free steel (TFS/ECCS), stainless steels rely exclusively on passive-film stability for corrosion protection, as no sacrificial metallic layer is present. Their corrosion behaviour is therefore controlled by a combination of metallurgical and surface-related factors, including alloying additions (Ni, Mo, and N), inclusion chemistry, grain-boundary character, heat-treatment-induced sensitization phenomena (e.g., chromium carbide precipitation), and the stability of the passive film under sterilization, cleaning agents, or chloride exposure [56,59].

This metallurgical framework confers exceptional chemical durability and long-term stability, which underpins the use of stainless steels in high-reliability and reusable packaging systems. At the same time, it imposes strict requirements on alloy selection, thermal processing, and surface condition, as sensitization or surface contamination can locally impair passivity and compromise corrosion resistance in service.

#### 2.4.2. Microstructure and Processing Routes

Austenitic stainless steels exhibit a face-centred cubic (FCC) crystal structure, which provides high ductility and formability even at modest thicknesses. This microstructural characteristic enables the fabrication of deep-drawn containers, pump housings, closures, and precision components requiring complex geometries without fracture. The FCC structure is also associated with a high strain-hardening capacity, which stabilizes wall deformation and delays localized necking under the biaxial stress states typical of packaging-related operations such as crimping, rolling, and seaming [56,57].

Ferritic stainless steels, characterized by a body-centred cubic (BCC) structure, display lower work-hardening rates and more pronounced planar anisotropy, which can limit drawability in complex shapes. Nevertheless, these alloys offer advantages in applications where high stiffness, magnetic responsiveness, and reduced alloying cost are prioritized, particularly when Ni-free solutions are preferred. Their use in packaging-related components is therefore generally restricted to geometries involving moderate deformation and controlled forming paths [58].

Processing routes for stainless steels employed in packaging applications typically include cold rolling, which refines grain structure and increases strength through work hardening, followed by annealing to restore ductility, control grain size, and mitigate residual stresses introduced during deformation. In addition, surface finishing—ranging from bright-annealed mirror finishes to mechanically polished or brushed surfaces—plays a critical role in defining passive film structure, surface energy, and interactions with food products, cleaning agents, or polymeric components [58,59].

During severe localized forming or assembly operations, such as threading, press-fitting, or crimping, stainless steels must maintain the integrity of the passive film. Although local disruption of the passive layer can occur under high contact stresses, rapid self-repassivation typically takes place in oxygenated environments. However, repeated abrasion or sliding contact—such as that experienced in pump actuators, valves, or reclosable mechanisms—may progressively expose microstructural heterogeneities or surface defects, influencing tribological behaviour and, in some cases, local corrosion susceptibility [57,59].

#### 2.4.3. Barrier and Mechanical Properties

Stainless steels provide intrinsically complete barrier properties as a consequence of their dense metallic lattice and the presence of a chemically stable passive film. As with other metallic packaging materials, they are fully impermeable to gases, vapour, and light, and their barrier performance does not rely on organic coatings or multilayer architectures. This intrinsic impermeability is retained under service conditions involving aggressive cleaning agents or repeated thermal exposure, where polymeric coatings or aluminum-based barrier systems may suffer degradation or loss of continuity [52,57].

From a mechanical perspective, stainless steels exhibit high stiffness and strength, with elastic moduli on the order of ~200 GPa and tensile strengths typically exceeding 250–300 MPa, depending on grade and processing condition. Combined with pronounced work-hardening behaviour—particularly in austenitic grades—these properties enable the fabrication of thin-walled components with good resistance to buckling, denting, and crack initiation under complex loading states [56,59].

In addition, stainless steels display excellent resistance to fatigue and thermal cycling, supporting reliable performance in applications subjected to repeated sterilization, cleaning-in-place (CIP), or sterilization-in-place (SIP) procedures. Mechanical integrity and barrier performance are generally preserved across multiple thermal cycles, provided that surface condition and passivation are maintained and that localized corrosion phenomena are avoided [57].

Overall, the combination of coating-independent barrier integrity and robust mechanical stability distinguishes stainless steels from tinplate, TFS/ECCS, and aluminum alloys in durability-oriented packaging contexts. These attributes underpin their selection in applications where long service life, repeated reuse, and resistance to harsh chemical or thermal environments are prioritized over lightweighting or high-volume formability.

#### 2.4.4. Surface Chemistry, Corrosion Behaviour, and Interactions with Coatings or Products

Corrosion resistance in stainless steel packaging systems is governed by the chemistry, continuity, and defect structure of the Cr_2_O_3_-based passive film. These include acidic food formulations, chloride-containing cosmetic products, alcohol-based or surfactant-rich pharmaceutical liquids, as well as repeated sterilization cycles involving steam, moist heat, or oxidizing agents. The ability of the passive film to adapt dynamically to such conditions underpins the suitability of stainless steels for chemically demanding and hygiene-critical packaging components [57,58].

In pharmaceutical and cosmetic packaging systems, stainless steels minimize metal ion release, thereby reducing the risk of product instability, colour change, or pH modification. Experimental studies on food-contact and pharmaceutical environments indicate that molybdenum-containing austenitic grades, such as AISI 316, provide enhanced resistance to pitting and crevice corrosion, particularly in chloride-rich or saline formulations, compared with Mo-free grades such as AISI 304 [56,59].

Despite their high intrinsic corrosion resistance, stainless steels are not immune to localized degradation. The stability of the passive film may be challenged under specific conditions, including elevated chloride concentrations, reducing chemical environments, repeated steam sterilization, aggressive cleaning-in-place (CIP) agents, and surface abrasion associated with mechanical actuation. Under such circumstances, corrosion initiation is strongly influenced by surface-related factors rather than by bulk alloy composition alone [57].

Studies addressing reusable containers and dispensing systems emphasize the critical role of surface condition, showing that parameters such as surface roughness, inclusions, polishing marks, heat tint, or residual contaminants can locally impair passivity and promote corrosion under stagnant or low-oxygen conditions. These scenarios are particularly relevant in pump valves, narrow channels, threaded closures, and complex dispensing geometries, where oxygen renewal is limited and mechanical wear may occur [20,58,59].

Unlike tinplate or tin-free steel (TFS/ECCS), stainless steels are typically used without internal metallic or organic coatings, and their performance relies primarily on appropriate alloy selection, surface finishing, and maintenance of passivity. Coatings are introduced only when specific functional requirements—such as reduced friction, controlled wettability, or enhanced wear resistance—are imposed, for example, in pharmaceutical pumps, reclosable dispensing systems, or moving components. In these cases, the adhesion of polymeric elements is governed by passive-film chemistry, surface energy, and the presence of adsorbed species, rather than by sacrificial or barrier-layer mechanisms.

#### 2.4.5. Mechanical, Sealing, and Functional Performance

Stainless steels are employed in packaging systems where mechanical reliability, dimensional stability, and long-term functional integrity are required to a degree that exceeds the capabilities of tinplate, tin-free steel, or aluminum-based solutions. Their performance arises from a combination of high yield strength, pronounced work-hardening behaviour, and stable surface passivation, which together support demanding service conditions [52,56].

From a mechanical standpoint, austenitic stainless steels provide resistance to crimping and mechanical sealing stresses, cyclic loading in reusable systems, deformation under pressurization or vacuum, and fatigue in actuator components such as springs and valves. The ability to sustain repeated deformation without crack initiation is particularly important in dispensing mechanisms and closures subjected to thousands of actuation cycles over the product lifetime [20,57]. Ferritic grades, while less ductile, offer higher stiffness and more predictable elastic–plastic response, making them suitable for rigid housings, structural components, and systems requiring magnetic handling or sensing [58].

Sealing performance in stainless-steel packaging systems differs fundamentally from that of tinplate or aluminum. Owing to the limited low-stress plastic deformation of stainless steels, sealing is typically achieved through high-precision mechanical interfaces—including threads, crimped joints, and press fits—or through the use of polymeric gaskets and elastomeric sealing elements, rather than through metal-to-metal conformability. As a result, dimensional tolerances, surface finish, and assembly precision become critical parameters, particularly in pharmaceutical containers with stainless-steel closures and in high-pressure cosmetic or medical dispensing systems [52,58].

In terms of functional properties, stainless steels also maintain full barrier performance against gases, vapour, and light, combined with chemical inertness compatible with stringent food, cosmetic, and pharmaceutical regulations. Their high thermal stability enables exposure to autoclave sterilization, dry-heat cycles, and, where required, radiation-based treatments without degradation of mechanical properties or loss of dimensional accuracy [57,59]. These characteristics underpin the use of stainless steels in reusable and refillable packaging systems, where durability over multiple use cycles is a primary design requirement [10].

In more specialized contexts, including electronic or hybrid packaging architectures, stainless-steel grades and specialty alloys may be selected for their dimensional stability, electromagnetic shielding capability, and reliable performance at mechanical joints or welded interfaces [52]. However, such applications remain limited in scope and volume.

Overall, stainless steels provide a combination of durability, chemical inertness, and mechanical reliability under repeated or aggressive service conditions. These advantages are accompanied by higher material cost and greater processing complexity, confining their use to applications in which stringent functional, safety, or reuse requirements justify their selection [52,57].

#### 2.4.6. Packaging Applications and Suitability—Stainless Steels and Specialty Alloys

Stainless steels occupy a niche but performance-critical role in packaging, being selected when chemical neutrality, long-term durability, cleanability, and dimensional stability outweigh lightweighting and high-throughput formability. In practice, this positioning favours reusable and high-reliability packaging architectures, where functional performance is governed primarily by passivation stability and surface condition rather than by sacrificial protection or coating-dependent barrier concepts [56,57].

In the food and beverage sector, stainless steels are mainly adopted in reusable containers and premium packaging systems where long shelf-life stability and repeated cleaning cycles are relevant. A representative example is provided by stainless-steel bottles for extra virgin olive oil, which showed improved storage performance under simulated retail light exposure compared with glass formats [10]. In reusable beverage containers, stainless steel also offers a clear advantage with respect to the absence of BPA-related migrants, in contrast to epoxy-lined aluminum bottles, reinforcing its suitability when organic contaminants from coatings are a concern [20]. Where acidic or chloride-containing formulations are involved, the suitability of stainless steels becomes grade- and surface-condition dependent; corrosion resistance and metal release remain generally low under standardized food-contact conditions, while localized corrosion risks increase in more aggressive environments, motivating conservative grade selection and strict surface finishing [56,59].

To extend the comparison to corrosion-resistant steel systems that do not rely on metallic overlays or coating-dependent protection, Table 4 summarizes the main stainless-steel grades used in packaging applications. Unlike tinplate and TFS, stainless steels provide intrinsic corrosion resistance through alloy composition, eliminating the need for sacrificial layers or mandatory internal lacquers in most food-contact applications.

As shown in Table 4, stainless steels differ fundamentally from tinplate and TFS systems in that corrosion resistance is intrinsically provided by alloy composition rather than by surface-engineered metallic or organic layers. This intrinsic protection concept explains their suitability for aggressive food and pharmaceutical environments, albeit typically at higher material cost and thickness compared with conventional steel-based packaging systems.

### 2.5. Metal–Matrix Composites (MMCs)

Metal–matrix composites (MMCs) represent the most specialized class of metallic materials addressed in this review, with applications confined almost exclusively to electronic, optoelectronic, and high-power device packaging. In these systems, the term packaging refers not to containment of consumer products, but to the thermo-mechanical protection, interconnection, and long-term reliability of functional devices, where thermal management, dimensional stability, and resistance to cyclic loading are critical performance drivers [12,60].

Unlike aluminum alloys or steel-based packaging materials, MMCs are not employed in mass-market containers. Their relevance arises in advanced packaging architectures—such as baseplates, heat spreaders, housings, and module substrates—where combinations of high thermal conductivity, tailored coefficient of thermal expansion (CTE), elevated stiffness, and reduced creep cannot be achieved with monolithic metals alone [13,61,62]. In the architectural framework adopted in this review (Figure 7f), MMCs therefore occupy the domain of functional and structural metallic packaging for electronic devices, distinct from container-based packaging materials.

The MMC systems most frequently reported in the literature for packaging-related functions include Al–SiC, Al–diamond, and Cu–SiC composites, which extend the performance envelope of aluminum and copper by enabling CTE matching with semiconductor materials (Si, GaN, and SiC) and improved resistance to thermo-mechanical fatigue under high heat-flux operation [34,35]. As a result, MMCs play a critical role in high-reliability electronic packaging, where the package itself acts as a structural and thermal component of the device.

#### 2.5.1. Composition and Metallurgical Architecture

The MMCs most commonly applied in packaging-related functions are based on aluminum or copper matrices reinforced with ceramic or carbon phases, designed to deliver tailored combinations of thermal conductivity, coefficient of thermal expansion (CTE), and mechanical stiffness required in electronic and power-device packaging.

Al–SiC composites typically employ SiC particles or preforms with reinforcement fractions in the range of 50–75 vol%, enabling low and controllable CTE values (~7–10 × 10^−6^ K^−1^) compatible with silicon and ceramic substrates used in microelectronic devices [61,62]. These systems represent one of the most mature MMC architectures for electronic packaging, balancing dimensional stability with manufacturability.

Al–diamond composites exploit the exceptionally high thermal conductivity of diamond, using powders or surface-treated diamond particles to achieve effective thermal conductivities in excess of 500–600 W·m^−1^·K^−1^, making them suitable for high heat-flux applications where conventional aluminum alloys are inadequate [34,35].

From a metallurgical standpoint, MMCs exhibit an inherently biphasic architecture, in which a ductile metallic matrix provides machinability and supports interfacial bonding and sealing, while the rigid reinforcement network governs stiffness, thermal conductivity, and thermal expansion. A central challenge in MMC design for packaging applications is the achievement of a chemically and mechanically stable matrix–reinforcement interface, free from excessive intermetallic formation, voids, or brittle reaction layers, which can degrade both thermal performance and long-term reliability [30].

#### 2.5.2. Microstructure and Processing Routes

The microstructure of MMCs used in electronic packaging is governed by the fabrication route, which determines reinforcement distribution, interfacial chemistry, and the presence of defects such as porosity or clustering. The processing approaches most commonly reported for packaging-related MMCs include infiltration techniques, powder-metallurgy routes, and laser-based fabrication processes.

Low-pressure infiltration, described extensively by [34], enables the production of dense Al–diamond composites with uniform reinforcement distribution and controlled formation of interfacial carbide layers. By regulating infiltration parameters and surface treatments, this approach allows optimization of thermal conductivity while limiting the formation of brittle reaction products that can compromise reliability.

Similarly, squeeze casting and pressure infiltration are widely employed for Al–SiC composites, yielding high relative density and reproducible CTE values when reinforcement morphology, volume fraction, and wetting behaviour are carefully controlled [62]. These processes are particularly suited to baseplate and heat-spreader geometries, where dimensional stability and thermal uniformity are critical.

More recent developments demonstrate the potential of laser cladding and laser-based deposition techniques to fabricate MMC layers directly on aluminum substrates with designed thermal-flow and stress-distribution geometries. As shown by Huang (2024) [35], this approach enables the local integration of MMCs into packaging housings without the need for fully consolidated bulk composite parts, offering additional design flexibility for advanced electronic packaging.

Across all processing routes, microstructural continuity remains essential. Porosity, reinforcement clustering, or weak matrix–reinforcement bonding reduce effective thermal conductivity and degrade resistance to thermo-mechanical fatigue, directly impacting the long-term reliability of electronic packaging modules subjected to high heat flux and cyclic loading.

#### 2.5.3. Barrier and Mechanical Properties

Metal–matrix composites exhibit barrier performance equivalent to that of their metallic matrices, characterized by near-zero permeability to gases and moisture and complete opacity to light. As in monolithic aluminum or copper systems, barrier integrity is not governed by the reinforcement phase but by the continuity of the metallic matrix, making MMCs inherently suitable for packaging functions requiring environmental isolation of sensitive electronic components.

Where MMCs differ fundamentally from conventional metals is in their thermal and mechanical response. Al–diamond composites achieve effective thermal conductivities exceeding 500 W·m^−1^·K^−1^, enabling rapid heat dissipation under high heat-flux conditions typical of power and optoelectronic devices [34,35]. In contrast, Al–SiC systems offer tailorable coefficients of thermal expansion, allowing close CTE matching with silicon, GaN, SiC, or ceramic substrates, thereby limiting thermally induced stresses at bonded and soldered interfaces [61,62].

From a mechanical standpoint, MMCs combine high elastic modulus, reduced creep, and enhanced resistance to thermo-mechanical fatigue, particularly under cyclic thermal loading. The presence of a rigid reinforcement network constrains matrix deformation, improving dimensional stability and preserving sealing-plane flatness in baseplates, heat spreaders, and structural packaging elements subjected to repeated temperature excursions [13,60].

As a result, the combination of intrinsic barrier integrity with engineered thermal and mechanical properties positions MMCs as indispensable materials in high-reliability electronic packaging, where failure is often governed by interfacial fatigue, warpage, or loss of thermal contact rather than by classical corrosion or permeability mechanisms.

#### 2.5.4. Surface Chemistry, Coating Interactions, and Corrosion Behaviour

The corrosion behaviour of MMCs differs fundamentally from that of monolithic aluminum or copper, owing to the presence of galvanically distinct reinforcement phases and heterogeneous matrix–reinforcement interfaces. In aluminum-matrix MMCs—such as SiC/Al systems—the matrix retains its natural oxide-forming tendency; however, local discontinuities in surface films and interfacial heterogeneities can promote micro-galvanic coupling under humid or electrochemically active conditions. This effect is most relevant in packaging assemblies with multi-material interfaces and joined stacks (e.g., metallizations, solders, adhesives/underfills, and local dissimilar-metal contacts), where localized electrolyte formation and potential differences can develop [63].

In electronic packaging applications, surface modification plays a decisive role in controlling interfacial chemistry and functional compatibility. Nickel, Ni–P, or Au metallizations are commonly employed to stabilize surface chemistry and improve solderability, ensuring predictable bonding to polymers, die-attach materials, and sealing glasses [12,60]. These surface treatments act primarily as interface-engineering layers, rather than classical corrosion barriers, tailored to the requirements of electronic assembly and sealing processes.

Diamond-reinforced MMCs present additional challenges related to wettability and interfacial stability. Tailored interlayers or coatings—such as WC, Mo, or Ti-based layers—are required to promote matrix–reinforcement adhesion and to suppress excessive formation of Al_4_C_3_, a brittle and moisture-sensitive phase that can compromise thermal conductivity, interfacial integrity, and long-term durability if not adequately controlled [30].

Because electronic packaging environments involve humidity exposure, thermal cycling, and complex interfacial stacks—including solders, adhesives, underfills, and glass seals—the long-term performance of MMCs is governed by the stability of passive surface layers, control of interfacial diffusion phenomena, and compatibility with metallization schemes used during bonding and sealing [60,64]. In this context, surface chemistry and interface design directly control the retention of thermal performance and mechanical integrity throughout the service life of MMC-based packaging components.

#### 2.5.5. Mechanical, Sealing, and Functional Performance

The functional requirements of MMCs in packaging differ fundamentally from those of conventional can-making metals. In MMC-based systems, thermal, dimensional, and mechanical stability under thermal and thermo-mechanical cycling constitute the primary performance metrics, reflecting the role of the package as an active mechanical and thermal element within electronic devices. From a mechanical standpoint, the high stiffness and elastic modulus of MMCs significantly limit deformation under clamping loads, internal stresses, and pressure fluctuations. This behaviour preserves sealing-plane flatness and mitigates bending-induced stress accumulation in solder joints and bonded interfaces. In composites with high reinforcement fractions, creep is strongly suppressed, and resistance to fatigue under high-temperature cycling is substantially improved, enhancing long-term reliability in power and optoelectronic modules [13].

Sealing behaviour in MMC-based packaging differs markedly from that of aluminum or steel containers. Rather than relying on plastic deformation to form hermetic seams, sealing is achieved through precision interfaces and engineered joints, including metal-to-metal contacts, bonded interfaces, and soldered or sintered joints. In this framework, the CTE matching capability of MMCs limits thermally induced interfacial stresses during service, supporting joint integrity under repeated thermal cycling [12].

These functional attributes enable MMC-based packages to operate reliably under thermal shock and rapid cycling conditions, where simultaneous control of heat flow, dimensional stability, and mechanical integrity is required. As demonstrated by Perron (2017) [65], the use of MMC baseplates enables substantial weight reduction while maintaining or improving thermal performance in aeronautic electronic packaging, highlighting the combined structural and thermal benefits of these materials.

Overall, MMCs occupy a highly specialized position within metallic packaging technologies, addressing performance requirements that emerge primarily in advanced electronic and thermal packaging architectures. Their use is therefore justified in applications where combinations of thermal conductivity, mechanical stiffness, and controlled thermal expansion are required under severe thermo-mechanical loading conditions.

#### 2.5.6. Packaging Applications and Suitability—Metal–Matrix Composites (MMCs)

Metal–matrix composites fulfil packaging functions almost exclusively in high-performance electronic and optoelectronic systems, rather than in food or consumer-goods applications. In electronic and optoelectronic device packaging, MMCs are adopted to balance the requirements for engineered thermal conductivity, controlled thermal expansion, and high stiffness, enabling packaging elements to dissipate heat while preserving dimensional stability. Typical MMC-based packaging components include heat spreaders, baseplates, chip carriers, multichip module lids, and thermal-management housings, where dimensional stability and heat dissipation under thermal and thermo-mechanical cycling are critical [12,60,66].

Al–SiC composites are widely employed to provide CTE matching with silicon and ceramic substrates, ensuring stability under thermal cycling and reducing interfacial stresses [13,61,62]. Al–diamond systems are selected for high heat-flux devices, where exceptionally high thermal conductivity is required to maintain junction temperatures within safe operating limits [30,34,35]. Cu-based MMCs are used in power-electronics modules when preservation of joint integrity and mitigation of thermo-mechanical fatigue dominate design requirements [12,60].

The suitability of MMCs is inherently constrained by high material cost and manufacturing complexity compared with monolithic aluminum or steel [13,60]. Consequently, MMCs are not positioned as general-purpose packaging materials, but remain confined to specialized electronic packaging domains where thermal loads, dimensional constraints, and long-term reliability requirements exceed the capabilities of conventional metallic systems [12,65].

To provide a structured overview of MMC architectures relevant to electronic and high-power device packaging, Table 5 summarizes representative systems by linking matrix–reinforcement design, processing route, key thermo-mechanical functions and typical packaging components. The comparison emphasizes that MMC performance is governed by the combined control of thermal conductivity, CTE matching and interface stability rather than by container-related requirements.

As shown in Table 5, MMCs enable packaging components to combine high thermal conductivity with controlled thermal expansion and enhanced stiffness, addressing failure drivers typical of electronic packaging—such as interfacial fatigue, warpage and loss of thermal contact—rather than container-related corrosion or permeability constraints.

### 2.6. Complementary Metallic Packaging Architectures

This section complements Section 2.1, Section 2.2, Section 2.3, Section 2.4 and Section 2.5 by clarifying additional ways in which metals contribute to packaging beyond the main material families. It distinguishes fully metallic containment, hybrid laminate architectures, and functional metal integration (coatings/fillers) to avoid ambiguity in the use of the term “metal packaging”. A comparable system-level perspective has recently been applied to packaging glass, reinforcing the importance of integrating material families within architecture-oriented and sustainability-driven analytical frameworks [67]. In particular, two complementary categories are considered. First, fully metallic packaging systems are briefly examined to acknowledge industrial and specialized applications in which the metal itself provides containment, mechanical protection, and durability without reliance on coatings or multilayer barriers. Second, metal-based coatings and metallic fillers are discussed as function-enabling strategies in polymeric or hybrid packaging systems, where metallic phases contribute specific functionalities—such as antimicrobial activity, sensing, or barrier enhancement—without defining the structural architecture of the package.

These complementary perspectives are not intended to extend the quantitative comparison developed later in this section, nor to redefine the application-driven selection framework established on the basis of real performance data. Instead, they serve to delimit the scope of the analysis and to avoid ambiguity in the use of the term “metal packaging”, which in the literature may refer to structurally metallic containers, metal-coated systems, or hybrid architectures with dispersed metallic phases.

By explicitly distinguishing these contributions, Section 2.6 provides a coherent bridge between the material-focused discussion of metallic packaging families and the application-level comparison and synthesis that follow, ensuring that all major modes of metallic participation in packaging are clearly identified before the final comparative assessment.

**Figure 8:**
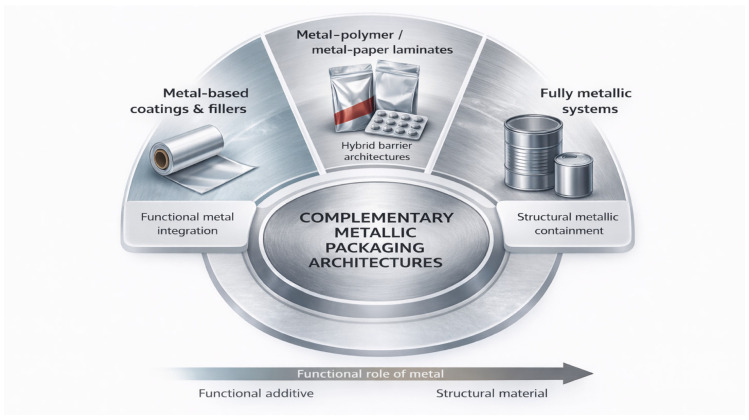
Conceptual overview of complementary metallic packaging architectures, highlighting the progressive transition from functional metal integration to fully structural metallic containment.

The different ways in which metals contribute to packaging performance beyond monolithic containers are schematically summarized in Figure 8, which distinguishes structural metallic systems, hybrid barrier architectures, and functional metal integration according to their architectural role rather than material family.

#### 2.6.1. Fully Metallic Packaging Systems

Fully metallic packaging systems represent a distinct and well-established category within the broader packaging landscape, in which containment, mechanical protection, and long-term durability are provided directly by the metallic material itself, without reliance on organic coatings, polymeric liners, or multilayer barrier architectures. This category is functionally distinct from the coated and multilayer metal packaging solutions predominantly discussed in the food- and consumer-oriented literature [2,68,69,70].

Typical examples include steel drums, barrels, and rigid metallic containers used for the storage and transport of bulk chemicals, petroleum-derived products, paints, solvents, and other regulated goods. In these applications, packaging performance is governed by structural parameters such as wall thickness, material strength, and geometric reinforcement (e.g., chimes, ribs, and closures), as well as by leak tightness and resistance to mechanical damage during handling and transport [68,69].

From a systems and service perspective, fully metallic packaging systems are closely associated with reuse, inspection, and reconditioning practices. Industrial drum management commonly involves cleaning and refurbishment operations that enable repeated use over extended service lifetimes, positioning these containers as durable assets within industrial supply chains rather than as single-use packages [70,71].

A defining feature of fully metallic packaging systems is their strong linkage to regulatory and logistical frameworks, particularly in the transport of dangerous goods. Container geometry, closure concepts, and allowable thickness reductions are therefore addressed within certification-driven requirements, with design optimization framed around compliance with drop, stacking, and leakproofness tests defined by transport regulations and technical standards [68,69].

Fully metallic packaging systems are also employed in highly specialized domains such as radioactive waste management, where waste packages are treated as engineered units for conditioning, transport, storage, and disposal. In this context, metallic containers—often in the form of drums or canisters—must maintain containment integrity and mechanical stability under normal and incident conditions, with performance verified through a combination of process knowledge and non-destructive or destructive characterization techniques [72].

Despite their importance in industrial logistics and specialized technical fields, fully metallic packaging systems occupy a functionally separate domain from the product packaging architectures analyzed in Section 2.1, Section 2.2, Section 2.3, Section 2.4 and Section 2.5. Their treatment here is therefore complementary and intended to delimit the scope, rather than to extend the comparative, application-driven framework developed later in this chapter [2,69,70,71].

#### 2.6.2. Metal–Polymer and Metal–Paper Laminates (Hybrid Barrier Architectures)

Metal–polymer and metal–paper laminates constitute a distinct class of hybrid packaging architectures in which a thin metallic layer—most commonly aluminum foil—acts as a barrier core, while polymeric and/or paper layers provide sealability, mechanical handling strength, printability, and processability. Unlike monolithic metallic containers, where the metal simultaneously defines structural integrity and barrier performance, laminated systems rely on distributed functionality, with performance emerging from multilayer integrity and interface design rather than from the metallic component alone [39]. In parallel, as a manufacturing analogue, multilayer metallic sheets and bimetallic laminates have been investigated as engineered architectures to combine dissimilar metallic functionalities within a single deformable substrate, typically relying on joining routes such as explosive welding and asymmetric rolling and on controlled forming to limit delamination and interfacial cracking [73,74]. In packaging-oriented multilayer systems, similar architecture-driven concepts apply to metal–polymer laminates, where barrier performance and durability depend primarily on interfacial integrity and end-of-life separability rather than on the metallic layer alone [75]. In these architectures, the metallic foil is embedded within a multilayer stack composed of sealant polymers, tie layers or adhesives, and external polymeric or fibrous supports. This configuration enables very high barrier performance at minimal material usage, but functional reliability is governed primarily by system-level integrity, including interfacial adhesion, defect tolerance, and resistance to mechanical damage during conversion and use [39]. As a consequence, the metallic foil should be interpreted as a functional layer within an interfacial stack rather than as a load-bearing substrate comparable to the metal families discussed in Section 2.1, Section 2.2, Section 2.3, Section 2.4 and Section 2.5.

From a mechanical and durability standpoint, laminated metal-based systems exhibit characteristic failure modes that differentiate them from bulk metallic packaging. Barrier degradation is frequently associated with flex cracking and pinhole formation in the foil, together with interfacial debonding under cyclic bending, thermal exposure, or localized stresses generated during sealing and opening operations. Recent experimental–numerical studies on coupled paper–aluminum laminates confirm that stress localization and damage initiation depend strongly on layer sequence, thickness ratios, and interfacial properties, supporting an architecture-driven interpretation of performance [76].

The multilayer nature of these systems also defines their end-of-life limitations. While aluminum and steel packaging benefit from mature recycling infrastructures, laminated structures pose intrinsic challenges to material recovery because dissimilar layers are intimately bonded and often poorly separable in existing waste streams. Thermal disengagement routes have been proposed and experimentally demonstrated for polymer-laminated aluminum packaging, enabling recovery of aluminum together with carbonaceous coproducts, while highlighting the strong sensitivity of recycled-metal quality to laminate composition, time–temperature conditions, and atmosphere control [77,78]. In practice, these constraints make laminated metal-based packaging less compatible with true closed-loop metallic recycling and more likely to follow secondary pathways or downcycling routes [75].

For these reasons, metal–polymer and metal–paper laminates are included here as complementary hybrid architectures to complete the landscape of metal-based packaging systems. They are not treated as a primary material family within the comparative framework of Section 2.1, Section 2.2, Section 2.3, Section 2.4 and Section 2.5, since their functional performance, durability, and recyclability are governed predominantly by multilayer design, interfacial engineering, and separability constraints rather than by the metallic substrate alone [75,76].

#### 2.6.3. Metallic Fillers and Metal-Based Coatings in Packaging Systems

In contrast to fully metallic packaging systems, a large body of contemporary literature addresses the incorporation of metallic elements into packaging materials through coatings, surface treatments, or dispersed fillers. In these architectures, metals do not provide structural containment but instead introduce targeted functional properties—such as antimicrobial activity, gas scavenging, optical response, or barrier enhancement—within polymeric or hybrid multilayer systems [2,79].

Metal-filled coatings and nanocomposites are predominantly investigated in the context of food and active packaging, where thin functional layers are used to extend shelf life, improve food safety, or enable intelligent responses to environmental stimuli. Common approaches include the incorporation of metallic nanoparticles (e.g., Ag, Au, Cu, ZnO, and TiO_2_) into polymer matrices or coating formulations, exploiting their high surface-to-volume ratio and surface reactivity to impart antimicrobial or antioxidant functionality [79,80].

Among metallic fillers, gold nanoparticles have received particular attention due to their chemical stability, tunable surface chemistry, and potential compatibility with food-contact applications. Reviews by Paidari et al. (2021) [81] and Ahari et al. (2022) [80] highlight the use of Au nanoparticles in active packaging systems to suppress microbial growth, scavenge reactive species, and act as sensing elements, while also emphasizing the need to control migration, particle size, and synthesis routes to address safety and regulatory concerns.

More broadly, metal-based coatings in packaging are often designed as ultra-thin functional layers deposited on polymer films, paper, or metallic substrates. Their performance depends critically on dispersion quality, interfacial adhesion, and long-term stability under humidity, temperature fluctuations, and mechanical deformation. Unlike monolithic metals, where barrier performance is intrinsic, the effectiveness of metal-filled coatings is inherently dependent on coating integrity and processing control, making these systems more sensitive to defects and ageing phenomena [2,82].

It is important to note that, despite frequent references to “metal-based packaging” in this literature, these systems differ fundamentally from the metallic packaging families discussed in Section 2.1, Section 2.2, Section 2.3, Section 2.4 and Section 2.5. Here, the metal does not define the packaging architecture but instead modifies the behaviour of an underlying polymeric or multilayer system. As a result, their performance, recyclability, and end-of-life pathways are governed primarily by the host material rather than by the metallic phase itself [2].

Within the scope of this review, metal-filled coatings and metallic fillers are therefore treated as function-enabling strategies, complementary to structural metallic packaging systems. Their inclusion highlights how metallic functionality can be decoupled from structural containment, enabling lightweight, multifunctional packaging solutions while simultaneously introducing new challenges related to migration, regulatory compliance, and circularity that differ from those associated with monolithic or fully metallic packaging systems [80,82].

### 2.7. Application-Driven Selection of Metallic Packaging Systems

This section synthesizes the material-specific analyses presented in Section 2.1, Section 2.2, Section 2.3, Section 2.4 and Section 2.5 into an application-driven perspective, interpreting the consolidated outcomes reported in the literature to illustrate how different metallic packaging systems are found to be suitable for distinct functional requirements across food, beverage, pharmaceutical, cosmetic, and technical domains. Rather than reiterating intrinsic material properties, the focus is placed on system-level suitability, highlighting how metallic families occupy specific performance niches as a result of recurring application constraints and processing conditions.

#### 2.7.1. Food and Beverage Packaging

In food and beverage packaging, material selection is consistently shaped by the combined requirements of barrier integrity, mechanical robustness, process compatibility, and cost efficiency, under conditions that often include thermal processing, internal pressure, and long storage times.

Aluminum alloys have emerged as the dominant solution in beverage packaging, where lightweighting, high formability, and compatibility with drawn-and-ironed manufacturing routes enable thin-walled containers capable of sustaining carbonation pressures. Their low density and strain-hardening behaviour support high-volume production of two-piece cans, while internal coatings ensure compatibility with acidic or carbonated products. In food applications, aluminum systems remain widely used but exhibit greater sensitivity to product chemistry and processing severity, making coating design and formulation-specific compatibility central to their suitability.

Tinplate constitutes the reference material for thermally processed foods requiring high structural rigidity, predictable double-seaming behaviour, and tolerance to autoclave-based thermal sterilization cycles. The steel substrate provides dimensional stability under vacuum and thermal loads, while tailored coating systems allow adaptation to a wide range of food chemistries. Tin-free steel (TFS/ECCS) occupies a complementary role, particularly in lids and ends, where coating adhesion, surface hardness, and precision scoring are prioritized over sacrificial corrosion protection.

Overall, food and beverage applications illustrate a fundamental trade-off between lightweighting and intrinsic mechanical stability, as well as between intrinsic metallic barrier performance and coating-dependent functionality. The resulting material selection reflects a balance between processing efficiency, long-term reliability, and product-specific compatibility rather than a single dominant performance criterion.

#### 2.7.2. Pharmaceutical and Cosmetic Packaging

Pharmaceutical and cosmetic packaging imposes stricter requirements related to chemical inertness, control of extractables and leachables, sterilization compatibility, and long-term functional stability. In pharmaceutical applications, aluminum foil-based systems remain the reference solution for blister packaging, where very low permeability to gases and moisture is required to ensure product stability. In these configurations, aluminum acts as the primary barrier element within multilayer architectures designed for seal integrity, mechanical robustness during forming, and controlled opening behaviour.

Stainless steels are widely adopted for closures, pumps, dispensing systems, and reusable containers in both pharmaceutical and cosmetic packaging. Their selection is typically driven by the stability of the passive surface layer, resistance to aggressive formulations and cleaning agents, and durability under repeated washing and autoclave-based thermal sterilization cycles. In this context, lightweighting considerations are secondary to surface integrity, dimensional precision, and long-term reliability. Tinplate and TFS systems are used more selectively, typically in formats where coating systems provide full isolation of the metallic substrate from the packaged product and where robust forming and seam performance remain advantageous.

#### 2.7.3. Technical and Electronic Packaging

Technical and electronic packaging operates under a distinct set of selection criteria, where thermal management, dimensional stability, hermeticity, and reliability under thermal and thermo-mechanical cycling dominate over cost and high-volume manufacturability. Metal–matrix composites (MMCs) are among the enabling solutions in this domain, enabling tailored coefficients of thermal expansion, high stiffness, and engineered thermal conductivity for integration with electronic components, substrates, and semiconductor devices. Stainless steels and specialty alloys are also commonly used in technical housings and enclosures requiring mechanical precision, corrosion resistance, and, where needed, electromagnetic compatibility. In these applications, metallic materials function less as containers and more as structural and functional elements within joined stacks and sealed assemblies, marking a transition from conventional packaging toward advanced encapsulation systems.

#### 2.7.4. Cross-Application Interpretation of Selection Outcomes

Across all application domains, the material selections discussed above can be consistently interpreted in terms of recurring system-level considerations, rather than bulk material properties alone. These considerations emerge implicitly from the reviewed literature and include tolerance to processing variability, reliability of joining and sealing technologies, sensitivity to localized defects, and the extent to which functional performance depends on surface coatings or multilayer architectures. Systems characterized by high intrinsic robustness tend to accommodate greater variability in processing and service conditions, whereas coating-dependent solutions require stricter control of surface integrity and manufacturing precision.

At the material level, this interpretation does not introduce new selection rules but rationalizes why different metallic families consistently occupy distinct functional roles across application domains. Aluminum alloys, tinplate, stainless steels, foil-based systems, and metal–matrix composites each recur in specific contexts because they satisfy dominant system-level requirements that cannot be compensated for by improvements in isolated material properties. The resulting convergence reflects application-driven constraints rather than sector-specific prescriptions.

**Figure 9:**
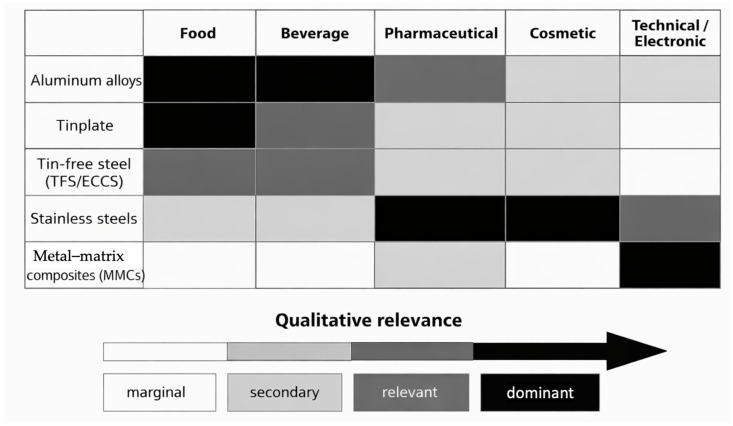
Application-driven interpretation map of metallic packaging systems. Qualitative matrix summarizing the consolidated relevance of major metallic packaging systems across different application domains. Rows represent material systems, while columns refer to packaging sectors. The grayscale scale indicates increasing application relevance—from marginal to dominant—based on a qualitative synthesis of the comparative analysis of metallic packaging systems discussed in the manuscript (including mechanical reliability, barrier performance, process compatibility, economic feasibility, and industrial maturity). The map does not represent quantitative performance metrics, weighted scoring, or numerical ranking, but provides an application-oriented overview to support interpretation of the selection outcomes discussed in Section 2.7.

The qualitative decision map reported in Figure 9 summarizes this application-driven interpretation, illustrating how no single metallic system is universally optimal. Instead, each material family occupies a defined functional niche shaped by the interaction between metallurgical architecture, surface engineering, processing routes, and service environment. The qualitative classification shown in the map derives from the comparative analysis of metallic packaging systems discussed throughout Section 2.1, Section 2.2, Section 2.3, Section 2.4, Section 2.5 and Section 2.6. The grayscale scale represents relative application relevance based on consolidated suitability outcomes and does not imply weighted scoring, numerical optimization, or quantitative ranking. This synthesis provides the conceptual foundation for the system-level structure–property relationships discussed in the following section.

## 3. Structure–Property Relationships in Metallic Packaging Systems

The performance of metallic packaging arises from the interplay among microstructure, surface chemistry, processing history, and thermo-mechanical response. Metallic materials encompass diverse crystalline architectures—including aluminum solid solutions, ferritic and austenitic steels, and reinforced metal–matrix composites—that give rise to markedly different mechanical behaviour, corrosion pathways, and functional properties in packaging applications.

**Figure 10:**
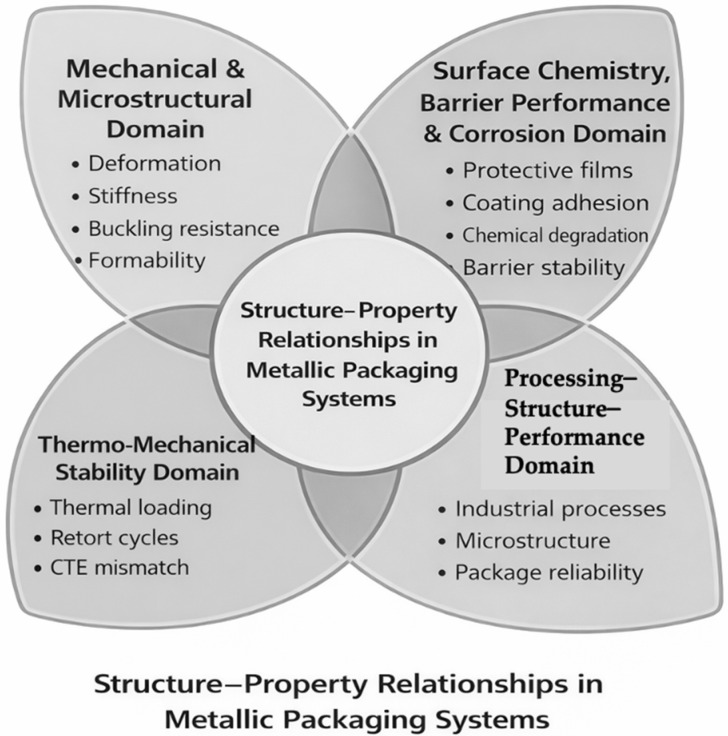
Four-domain conceptual model of structure–property relationships in metallic packaging systems. Schematic representation of the four interrelated mechanistic domains governing the performance of metallic packaging: (i) mechanical and microstructural behaviour, (ii) surface chemistry, barrier performance and corrosion mechanisms, (iii) thermo-mechanical stability under processing and service conditions, and (iv) processing–structure–performance coupling. The model provides an interpretative framework to rationalize how microstructure, surface state, thermal response, and manufacturing history collectively determine packaging functionality and reliability, without implying a material selection or optimization methodology.

To provide a unified interpretative framework, this section adopts a four-domain structure, conceptually represented as a four-petal model (Figure 10), which organizes the causal mechanisms linking metallic structure to packaging performance:

1. Mechanical and Microstructural Domain, governing deformation, stiffness, buckling resistance, and formability;
2. Surface Chemistry, Barrier Performance and Corrosion Domain, which determines the stability of protective films, coating adhesion, and resistance to chemical degradation;
3. Thermo-Mechanical Stability Domain, describing how metals respond to thermal loading, retort cycles, and coefficient-of-thermal-expansion (CTE) mismatch;
4. Processing–Structure–Performance Domain, highlighting the coupling between industrial processes and final package reliability.

This four-domain structure is introduced as an interpretative model to rationalize structure–property relationships in metallic packaging systems. The following sections examine each domain in detail. Section 3.5 then brings these insights together in a comparative discussion across the main metallic families.

### 3.1. Mechanical and Microstructural Behaviour

Mechanical response in metallic packaging is primarily governed by crystal structure, grain size, crystallographic texture, and processing-induced defects. Although aluminum, low-carbon steel, stainless steel, and metal–matrix composites may serve similar packaging functions, their mechanical properties arise from fundamentally different micromechanisms.

#### 3.1.1. Aluminum Alloys

Aluminum alloys used for cans, closures, and foils derive their behaviour from solid-solution strengthening and strain-hardening. Rolling and annealing generate strong Cube and Goss textures, producing anisotropic deep-drawing behaviour and controlled thinning during ironing. The relatively low Young’s modulus (~70 GPa) renders aluminum more susceptible to panel buckling than steel for comparable geometries, but its high strain-hardening rate and capacity for uniform elongation enable lightweight structures resistant to denting and collapse under internal pressure. Microstructural stability during retort cycles is crucial: recrystallization or excessive softening can impair top-load strength.

#### 3.1.2. Tinplate and Tin-Free Steel

In tinplate, the steel substrate dictates mechanical performance. Ferritic grains elongated during rolling provide high stiffness (E ≈ 200 GPa) and tensile strengths typically exceeding 400 MPa, enabling very thin walls without loss of structural integrity. Temper rolling increases work-hardening and controls flangeability during seaming. The presence of Fe–Sn intermetallics affects surface hardness and local deformation, especially in the hook and body-hook regions of a double seam.

TFS exhibits similar mechanical attributes, though the absence of a ductile tin layer increases friction during forming and may elevate localized strain near bends or localized joining features.

#### 3.1.3. Stainless Steels

Austenitic stainless steels (304, 316) exhibit high toughness, exceptional resistance to crack initiation, and superior fatigue performance. Their mechanical behaviour differs from ferritic steels due to slip on multiple systems and strain-induced martensite in certain grades. These features support applications requiring repeated sterilization, mechanical cycling, or high-dimensional precision.

#### 3.1.4. Metal–Matrix Composites

MMCs provide extreme stiffness, low creep, and high fatigue resistance. Reinforcements such as SiC or diamond raise the modulus into the 200–250 GPa range while maintaining low density. This microstructure–mechanical synergy enables enclosures and baseplates that remain dimensionally stable under thermal and mechanical loads encountered in electronics packaging.

#### 3.1.5. Cross-Material Considerations

Across all systems, microstructural features—grain morphology, texture, dislocation density, precipitation state, and interface architecture—define forming behaviour, buckling resistance, and failure modes. Metals exhibit a broad spectrum of mechanical anisotropies and local strain concentrations that must be managed to ensure seam integrity, wall stability, and long-term structural reliability.

### 3.2. Surface Chemistry, Barrier Performance and Corrosion Mechanisms

Barrier performance in metallic packaging is controlled not only by bulk impermeability but also by the properties of surface films, intermetallic layers, and organic coatings. Corrosion resistance is highly material-specific, dictated by thermodynamics, environment, and the stability of passivating layers.

#### 3.2.1. Aluminum

Aluminum forms a thin Al_2_O_3_ film that provides baseline corrosion protection. Hydration, pH, and temperature influence film stability. In the presence of acidic media or chloride ions, localized pitting can initiate, especially at coating defects [83]. Interactions with lactic and acetic acids, observed in certain fermented beverages, have been reported to promote corrosion during aluminum beverage can storage, with dissolved Al and visible liner degradation increasing with organic-acid content and decreasing pH; in this context, liner type may not prevent corrosion if aggressive chemistry is sustained [17]. Similar corrosion and migration phenomena have also been reported for aluminum cans exposed to alcoholic beverage matrices, where coating ageing and electrolyte permeation progressively reduce barrier performance and increase metal release [84]. Recent research has also explored the development of alternative bio-derived coating systems designed to replace BPA-based epoxy linings while maintaining corrosion protection in aggressive beverage environments [85].

#### 3.2.2. Tinplate

Tinplate performance in food packaging depends on the integrity of the organic coating system and the control of oxygen exposure during processing and storage. Small coating discontinuities can compromise barrier performance, particularly after thermal sterilization cycles, which impose severe mechanical and chemical stresses on the coating layer. Residual headspace oxygen is a key factor influencing internal package stability and long-term performance. In sulfur-containing foods, surface discoloration phenomena may occur, but when coating integrity is preserved these effects are generally considered aesthetic rather than structural [25].

#### 3.2.3. Tin-Free Steel

TFS relies almost entirely on its organic coating for corrosion protection. The chromium-based passive layer primarily promotes coating adhesion and inhibits underfilm delamination, while providing negligible sacrificial protection. As a result, corrosion at coating defects can propagate rapidly, making coating selection and curing particularly critical for acidic or low-pH food applications [4,41].

#### 3.2.4. Stainless Steels

Stainless steels exhibit superior chemical durability due to the Cr_2_O_3_ passive film, which is self-healing and stable across a broad pH range. Pitting may occur in chloride-rich environments, but Mo additions mitigate this effect. Stainless steels maintain barrier integrity under sterilization, cleaning cycles, and repeated mechanical handling, making them ideal for high-purity environments and reusable systems.

#### 3.2.5. Metal–Matrix Composites

MMCs maintain barrier performance similar to aluminum but may exhibit enhanced corrosion resistance depending on reinforcement chemistry and interface stability. Coatings (Ni-P, Au) are often applied for solderability in electronic packaging. Their resistance to coating delamination under thermal cycling exceeds that of monolithic metals.

#### 3.2.6. Key Mechanisms

Across all materials, failure of barrier performance is linked to localized film breakdown, interfacial defects, coating mis-cure, or galvanic coupling at discontinuities. Understanding these mechanisms is essential for predicting durability and preventing migration phenomena, which are treated in detail in Section 4.

### 3.3. Thermo-Mechanical Stability

Thermal exposure during processing (pasteurization, sterilization, and retort) and service conditions—during storage, transport, and use—impose mechanical and dimensional demands on metallic packaging. The focus is on thermo-mechanical mechanisms common to different metallic systems that govern dimensional stability and coating or joint integrity under thermal loading and cycling. Thermo-mechanical coupling arises from differential thermal expansion, stress relaxation, and fatigue damage.

#### 3.3.1. Aluminum

Aluminum’s relatively high coefficient of thermal expansion (23–24 × 10^−6^ K^−1^) makes it more susceptible to thermally induced dimensional changes under heat, particularly in constrained or thin-walled geometries. Retort cycles can soften strain-hardened alloys, reducing top-load strength. Mismatch between aluminum and lacquers may induce interfacial stresses, contributing to coating cracking or loss of adhesion.

#### 3.3.2. Tinplate and TFS

Steel’s lower CTE (~12 × 10^−6^ K^−1^) improves dimensional stability during thermal cycles. Tinplate undergoes minimal thermal distortion, though repeated retort cycles can promote tin dissolution and accelerate coating degradation. TFS benefits from stable chromium oxides that maintain coating adhesion under thermal load, making it suitable for high-temperature closures and processed foods.

#### 3.3.3. Stainless Steels

Stainless steels exhibit excellent thermal stability, with minimal microstructural evolution under sterilization. Their resistance to thermal shock and creep makes them suitable for reusable containers, pharmaceutical closures, and precision components.

#### 3.3.4. Metal–Matrix Composites

The most distinctive thermo-mechanical behaviour is observed in MMCs. Reinforcements such as SiC and diamond dramatically reduce the CTE (down to <10 × 10^−6^ K^−1^) while increasing thermal conductivity beyond 200–500 W/m·K. This combination suppresses thermal deformation and significantly delays thermo-mechanically induced fatigue cracking, essential for electronic packaging under rapid thermal cycling.

#### 3.3.5. Cross-Material Considerations

Dimensional stability depends on both intrinsic CTE and processing history. Residual stresses from forming or welding influence long-term stability. Thermal cycling can render some coatings brittle, alter passivation layers, and drive interfacial degradation. Understanding these coupled effects is crucial to predicting package longevity.

### 3.4. Processing–Structure–Performance Coupling

Manufacturing routes determine microstructural state, surface morphology, mechanical anisotropy, and final performance of metallic packaging. Processing effects are therefore inseparable from structure–property relationships. This section focuses on how industrial processing routes actively shape microstructure and interfaces, thereby governing the mechanical reliability and functional performance of metallic packages.

#### 3.4.1. Forming Operations

Deep drawing, ironing, embossing, and seaming impose complex strain paths. Aluminum exhibits benign strain distribution due to high work-hardening, whereas steels require controlled temper selection. In can-making, wall thinning, earing, and flange cracking reflect the interaction between crystallographic texture and forming stresses.

#### 3.4.2. Surface Finishing and Coating Adhesion

Surface roughness from rolling or temper finishing directly affects lacquer wetting and adhesion. In aluminum, conversion coatings modify surface chemistry to stabilize the oxide layer and promote interfacial bonding. In tinplate and TFS, coating adhesion depends on the continuity and hydration state of Fe–Sn intermetallics or Cr-oxides.

#### 3.4.3. Heat Treatment, Annealing, and Tempering

Annealing governs grain size, texture, and recrystallization, influencing drawability and buckling resistance. Temper rolling in tinplate and TFS controls yield strength and stiffness, enabling stable double seams and resistance to panel deformation.

#### 3.4.4. Joining and Sealing

Sealing performance depends on localized deformation in seams, rivets, and closures. Failure often initiates from microcracks or coating fractures generated during forming. In stainless steels and MMCs used for advanced closures, joining methods (welding, crimping, and brazing) must preserve microstructural integrity and avoid sensitization or interfacial degradation.

### 3.5. Comparative Evaluation Across Metallic Packaging Systems

The comparative assessment of metallic packaging materials synthesizes the structural–property relationships discussed in this section with the quantitative trends summarized in Table 6, which consolidates the representative ranges reported in Table 1, Table 2, Table 3, Table 4 and Table 5 into a single comparative overview. Aluminum, coated and uncoated steels, stainless steel, and advanced metal–matrix composites occupy distinct positions within a multi-criteria performance space defined by mechanical strength, barrier capacity, packaging weight, cost, recyclability, and environmental impact.

The data summarized in Table 6 provide an indicative, system-oriented comparison of representative metallic materials used in packaging, combining intrinsic material properties with performance-relevant attributes emerging from processing and surface engineering. The reported ranges do not represent absolute material limits but reflect typical values for packaging-grade alloys and composites, as commonly employed in industrial applications.

Rather than supporting direct material selection, the table serves to contextualize the structure–property relationships discussed in Section 3.1, Section 3.2, Section 3.3 and Section 3.4, highlighting how differences in crystal structure, surface chemistry, thermo-mechanical response, and processing sensitivity translate into distinct performance profiles. In particular, the comparison illustrates how mechanical robustness, barrier capacity, packaging weight, corrosion resistance, and recyclability are not independently optimized within a single metallic system but emerge from trade-offs inherent to each material family.

**Figure 11:**
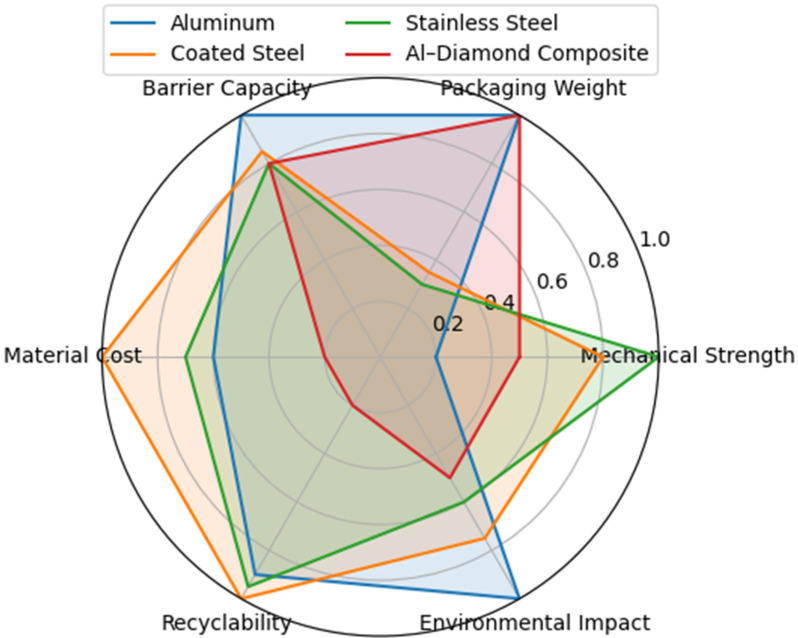
Comparative performance of metals and metal–matrix composites in packaging. Radar chart comparing aluminum, coated steel, stainless steel, and Al–diamond composites across six normalized criteria: mechanical strength, packaging weight, barrier capacity, material cost, recyclability, and environmental impact. Each axis is scaled from 0 to 1, with 1 indicating the best relative performance within the set of materials considered. The normalized scores are derived from the representative values summarized in Table 6 by applying a relative normalization within the compared set (best-performing material for each criterion = 1). The chart provides a qualitative, system-level comparison highlighting trade-offs among different metallic packaging families, rather than absolute performance metrics.

On this basis, the comparative evaluation developed in this section rationalizes why aluminum, coated steels, stainless steels, and metal–matrix composites occupy different regions of the multi-criteria performance space. These trends are subsequently synthesized in a normalized graphical form in Figure 11, which integrates mechanical, barrier, economic, and sustainability-related indicators into a unified qualitative comparison.

#### 3.5.1. Mechanical Properties

Coated and uncoated steels exhibit the highest structural strength among conventional metallic packaging materials, with tensile strengths typically in the range of 400–600 MPa and Young’s moduli exceeding 190 GPa. This combination provides high resistance to elastic deformation, buckling, and denting under mechanical loads encountered during handling, transport, and retort processing.

Aluminum alloys, while significantly lighter, display moderate strength levels (100–300 MPa) and a lower elastic modulus (70–80 GPa). Nevertheless, their pronounced strain-hardening behaviour and favourable forming response enable the production of deep-drawn and ironed containers with thin walls that remain mechanically stable under internal pressure and service loads.

Tin, employed primarily as a surface coating rather than as a structural material, exhibits comparatively low mechanical strength (≈220 MPa tensile strength, ≈50 GPa modulus). This confirms that its functional contribution in packaging is predominantly chemical and electrochemical, rather than load-bearing.

Metal–matrix composites (e.g., Al–diamond systems) extend the mechanical performance envelope beyond that of monolithic packaging metals. Their high stiffness (moduli exceeding 250 GPa) and enhanced fatigue resistance enable exceptional dimensional stability under combined mechanical and thermal loads. However, elevated cost and processing complexity restrict their application to specialized sectors such as electronic and thermally managed packaging.

#### 3.5.2. Barrier Performance

Aluminum and coated steel systems provide effective barriers against gases and moisture as a consequence of their intrinsic metallic impermeability, combined—where applicable—with continuous organic coatings. In particular, aluminum foil approaches near-total impermeability to oxygen and water vapour when free of pinholes, making it a reference material for high-barrier packaging applications.

In tinplate packaging, barrier capacity is governed by the integrity of the coating system rather than by the metallic substrate alone. As long as coating continuity is maintained, barrier performance remains stable; however, localized defects or coating discontinuities can expose the steel substrate, promoting underfilm corrosion that rapidly compromises protective function.

Metal–matrix composites do not provide intrinsic improvements in gas or moisture barrier properties relative to monolithic aluminum systems. Their contribution to packaging performance is instead indirect, arising from superior thermal conductivity and dimensional stability, which help preserve barrier integrity in applications involving thermal cycling, heat-sensitive formulations, or electronic housings.

#### 3.5.3. Recyclability

Aluminum and steel maintain fully established recycling infrastructures with high material recovery rates and minimal quality degradation across cycles. Tin presents no intrinsic recyclability barrier but complicates steel melting operations and requires dedicated management in recycling streams.

In contrast, Al–diamond and other MMCs are technically recyclable but lack large-scale industrial pathways; their hybrid nature hinders dismantling and separation, reducing economic feasibility.

#### 3.5.4. Environmental Impact

The use of recycled aluminum is associated with substantial reductions in energy demand and CO_2_ emissions relative to primary production, supporting low-impact packaging strategies when high collection and recycling rates are achieved. Steel also benefits from established recycling routes, aided by efficient magnetic separation and mature recovery infrastructures.

By contrast, metal–matrix composites typically involve higher embodied energy and less-defined end-of-life pathways. Their environmental balance is therefore more likely to be favourable in long-lifetime or reusable applications, where the functional benefits and service life can offset end-of-life limitations.

#### 3.5.5. Cost Efficiency

Steel generally exhibits lower material cost on a per-kilogram basis (typical raw material and industrial sheet prices near ~0.8–1.0 €/kg) compared with aluminum, whose base commodity price is nearer ~2.7–3.2 €/kg in early 2026. The cost of tin as a pure metal is substantially higher (on the order of tens of €/kg), but because it is used in very thin coatings, its contribution to the overall cost remains limited. Metal–matrix composites, involving high-cost reinforcements, occupy the upper end of material cost considerations.

The cost ranges reported in this section are intended as indicative orders of magnitude rather than precise industrial prices. They reflect typical market values for raw materials or standard packaging-grade semi-finished products and are subject to variation depending on alloy composition, product form (sheet, foil, and coating), regional market conditions, and temporal fluctuations. In particular, for coating materials such as tin, the quoted values refer to the base metal cost, while their actual contribution to packaging cost remains limited due to the extremely small quantities employed. These indicative comparisons are therefore used to support qualitative trends and relative positioning of metallic packaging systems, rather than detailed economic assessments.

#### 3.5.6. Packaging Weight

Aluminum is the lightest standard metallic material used in packaging (density ≈ 2.7 g/cm^3^), enabling substantial mass reductions relative to steel-based systems (density ≈ 7.85 g/cm^3^). Metal–matrix composites can achieve comparable or even lower effective densities depending on reinforcement content, while simultaneously offering high stiffness-to-weight ratios that are advantageous in applications requiring dimensional stability under combined mechanical and thermal loads.

To facilitate cross-material comparison, these quantitative and qualitative indicators are integrated into a normalized radar representation (Figure 11), where each axis reflects a key performance criterion derived from Table 6 and expressed on a relative scale within the set of materials considered.

#### 3.5.7. Interpretation of Radar Differences

The radar representation shown in Figure 11 provides a qualitative synthesis of the comparative trends discussed in this section, integrating mechanical, barrier-related, economic, and sustainability-oriented indicators into a unified graphical framework. Rather than identifying an optimal material, the chart highlights how different metallic systems occupy distinct regions of the multi-criteria performance space.

Aluminum exhibits high scores in packaging weight and recyclability, reflecting its low density and well-established recycling infrastructure. At the same time, its lower elastic modulus relative to steel shifts its mechanical-performance axis toward intermediate values, consistent with the microstructural mechanisms discussed in Section 3.1.

Coated steel systems achieve the highest scores in mechanical robustness and barrier capacity, owing to their high stiffness and structural strength combined with continuous coating systems. However, their higher density and transport-related impacts reduce relative performance along the weight-efficiency and environmental-impact axes, in agreement with the thermo-mechanical and corrosion-related considerations outlined in Section 3.2 and Section 3.3.

Metal–matrix composites appear as outliers within the radar representation. They reach peak values in stiffness and dimensional stability, but score lower in cost efficiency, recyclability, and environmental impact. This pattern reflects the inherent trade-off between advanced functional performance and the material complexity, energy intensity, and end-of-life challenges associated with these systems.

Overall, the radar comparison confirms that no single metallic material simultaneously optimizes all performance criteria. Instead, each system occupies a specific functional niche shaped by the interplay between its structural architecture, surface chemistry, processing routes, and service conditions.

Because several radar axes—including recyclability, environmental impact, and aspects related to migration safety—depend on regulatory frameworks and life-cycle assessment methodologies, their detailed quantitative treatment is deferred to Section 4 and Section 5.

Figure 9 and Figure 11 provide complementary but non-overlapping perspectives on metallic packaging systems. The application-driven selection map in Figure 9 summarizes the relative relevance of different metallic families across packaging sectors, whereas the comparative radar chart in Figure 11 rationalizes these application trends by linking them to underlying structure–property relationships and normalized performance indicators. Together, the two figures clarify why material choice in metallic packaging is inherently application-specific and cannot be reduced to a single dominant criterion.

## 4. Regulatory and Safety Framework for Metallic Packaging

The safety and regulatory landscape governing metallic packaging is shaped by the dual need to protect consumer health and to ensure the long-term stability of packaged products under a wide range of environmental and processing conditions. Regulatory requirements differ geographically but converge on the principle that metal–food or metal–pharmaceutical interactions must not compromise product quality, chemical safety, or functionality throughout the intended shelf life [86]. Metallic systems are prone to corrosion, ion migration, coating degradation, and thermo-mechanical ageing; compliance with regulatory standards is inseparable from material selection and surface-engineering strategies.

Recent reviews confirm that regulatory frameworks for food-contact packaging materials are increasingly driven by chemical migration concerns, regional regulatory divergence, and the need for harmonized safety assessment strategies across materials and application sectors [87]. The regulatory instruments and technical standards referenced in this section are summarized in Appendix A.

Table 7 provides a classification of the regulatory instruments and standards cited in this section, grouping them according to their functional role and regulatory context (European Union, United States, and international frameworks), and indicating their relevance across different application sectors.

### 4.1. Regulatory Architecture for Metals in Food, Pharmaceutical and Technical Packaging

#### 4.1.1. European Union Framework

In the EU, metallic packaging is regulated under Regulation (EC) 1935/2004, which establishes the requirement that materials in contact with food must not:

- Endanger human health;
- Cause an unacceptable change in food composition;
- Alter organoleptic characteristics.

Specific requirements for metals and coatings derive from:

- Commission Regulation (EU) 10/2011 (for polymeric coatings used on metals);
- EN 602:2004 and EN 10333 (for tinplate and TFS);
- EN 13130/EN 1186 for migration testing;
- EFSA guidelines on specific migration limits (SMLs) for aluminum, tin, chromium, nickel, and other alloying elements.

For metallic materials, Council of Europe technical guidelines are widely adopted as an operational reference for compliance assessment, defining specific release limits (SRLs) and harmonized simulant conditions for metals and alloys in food contact [56,88].

While specific migration limits (SMLs) are formally established within EFSA-based frameworks and polymer-related regulations, SRLs are commonly applied to metallic substrates to account for corrosion-driven release mechanisms not adequately described by polymer-oriented approaches [56].

Recent EFSA assessments have updated tolerable intake thresholds for aluminum, highlighting significant variability between national limits and calling for harmonization [15].

For steel-based systems, legislation focuses on coating continuity and corrosion resistance, as uncoated steel is not acceptable for direct-contact food packaging. Requirements on tinplate include control of free tin levels, Fe–Sn intermetallic stability, and the absence of organotin residues.

#### 4.1.2. United States and International Standards

The U.S. regulatory framework is governed by:

- FDA Title 21 CFR, which defines permitted materials and coating substances;
- NSF standards for reusable metallic containers;
- ANSI/ASTM protocols for corrosion and leaching tests.

Aluminum is permitted for direct food contact provided that migration levels remain within FDA-established limits. Epoxy, polyester, acrylic and BPA-NI coatings are regulated under substance-specific clearances requiring detailed toxicological evaluation.

Internationally, ISO standards provide harmonized methods for:

- Migration testing (ISO 4531 for ceramics extended to metals in some jurisdictions);
- Coating adhesion and curing (ISO 4624);
- Corrosion testing (ISO 9227, salt-spray simulations);
- Lacquer continuity (ISO 8301, EN 10333).

Pharmaceutical metal packaging must additionally comply with USP <661> and <671>, which address container integrity, surface reactivity, and permeation tests.

The main regulatory instruments and technical standards referenced in this section are summarized in Appendix A.

### 4.2. Migration Phenomena and Safety Assessment

It should be noted that test parameters such as pH, temperature, contact duration, and headspace composition are not arbitrary experimental choices, but are explicitly defined within regulatory testing protocols to represent worst-case exposure scenarios [88].

Migration from metallic packaging involves the release of metal ions or coating components into the product under thermal, mechanical, or chemical stress. The governing factors include pH, salinity, oxygen content, redox environment, temperature, and headspace composition.

#### 4.2.1. Metallic Ion Migration

##### Aluminum

Although aluminum forms a passivating Al_2_O_3_ layer, acidic beverages (lactic, citric, and acetic acids) can destabilize the film and initiate pitting, especially at coating discontinuities. Shukla (2023) and Sheehan (2025) [16,17] demonstrated measurable Al^3+^ migration in low-pH beverages, with local corrosion intensified by headspace oxygen and organic acids.

##### Tinplate and TFS

Tin can migrate in reducing environments, whereas steel dissolution occurs rapidly when coating defects expose the substrate. Wu (2024) [4] and Chang (2024) [29] confirmed that corrosion accelerates at weld seams or scratch lines, driven by oxygen ingress and sterilization cycles.

Chromium migration in TFS is typically minimal when the Cr/Cr-oxide duplex layer remains stable; however, regulatory restrictions on Cr(VI) demand strict process control and documentation, as these limitations directly affect material compliance rather than representing purely environmental constraints.

##### Stainless Steel

Stainless steels exhibit minimal migration under food-contact conditions when passive films remain intact. Nickel release is closely monitored, especially in acidic environments; USP and EU guidelines define strict SML values.

#### 4.2.2. Migration from Coatings and Functional Additives

Modern lacquers (epoxy, polyester, acrylic, organosols, and BPA-NI systems) include oligomers, additives, and catalysts that may migrate if curing is incomplete. Migration testing must be performed using worst-case simulants according to EN 1186.

Metal/metal-oxide nanoparticles (ZnO, TiO_2_, and Ag), explored for antimicrobial or active-barrier functions (Sani 2024) [89], introduce emerging concerns regarding:

- Nanoparticle detachment;
- Dissolution into ionic species;
- Aggregation and transport in food matrices.

Current regulations require case-by-case evaluation, and harmonized “nano-specific” migration limits have yet to exist.

In addition to conventional coating systems, silane-based composite coatings have been investigated as environmentally compatible corrosion-protection strategies offering significantly lower toxicity than traditional chromate-based treatments [90].

#### 4.2.3. Accelerated Testing and Predictive Assessment

Because real-time ageing is impractical, predictive tests are widely used:

- Electrochemical impedance spectroscopy (EIS) to monitor coating degradation and barrier performance of protective layers;
- Thermal cycling to simulate retort and storage conditions;
- pH cycling to evaluate susceptibility to episodic exposure;
- Salt-spray (ISO 9227) for corrosion benchmarking.

Ali (2025) [91] demonstrated that combined thermal–chemical–electrochemical protocols can simulate five years of shelf life in <60 days, enabling reliable pre-market assessment.

### 4.3. Safety Considerations in High-Value and Reusable Metallic Packaging

Although not specific to disposable food-contact packaging, hygienic and sanitary standards such as 3-A and NSF play a regulatory role for reusable, pharmaceutical, and high-value metallic packaging systems, where surface integrity, cleanability, and long-term chemical inertness are critical safety requirements [59].

Stainless steel, anodized aluminum and MMCs are used in pharmaceutical, medical, and electronic packaging requiring:

- High chemical inertness;
- Resistance to thermal sterilization;
- Repeatable sealing performance;
- Minimal particle shedding;
- Tight dimensional tolerances.

Engler (2025) [6] shows that reusable stainless-steel systems rely on controlled surface finishes (Ra < 0.4 µm), which improve cleanability and reduce ion release.

In electronics packaging, MMCs must meet strict criteria on solderability, outgassing, and interfacial stability to avoid delamination in thermal cycling.

These systems typically fall under stricter regulatory oversight (USP, ASTM, IEC, and ISO) and require comprehensive documentation covering materials, coatings, manufacturing, and sterilization compatibility.

### 4.4. Regulatory Trends and Outlook

Three trends are reshaping the regulatory framework for metallic packaging:

(1) Stricter controls on migration and coating composition

BPA restrictions, scrutiny of bisphenol analogues, and requirements for low-migration coatings (EU, FDA) push the industry toward polyester, acrylic, and hybrid organic–inorganic lacquers.

(2) Integration of circularity principles into material safety

The EU Green Deal and packaging regulations link recyclability, traceability, and purity of recyclate to market access. Digital material passports for coatings and surface treatments [59] are becoming central tools to ensure compatibility with recycling streams.

(3) Expansion of safety frameworks beyond food packaging

Electronics, pharmaceutical and active packaging systems require harmonized migration-free behaviour under:

- High-temperature sterilization;
- High-voltage electrical environments;
- Long-term storage;
- Repeated mechanical cycling.

A consolidated overview of the regulatory and standardization framework discussed in this section is provided in Appendix A.

Future work will emphasize life-cycle-integrated safety, combining chemical risk, recyclability, and durability in a unified assessment approach.

### 4.5. Concluding Remarks

Regulatory and safety considerations for metallic packaging are inseparable from material selection, microstructural stability, surface engineering, and processing history. The diversity of metallic systems—ranging from aluminum and tinplate to stainless steel and MMCs—requires tailored compliance strategies grounded in both mechanistic understanding and standardized testing.

The trends outlined above highlight a shift toward safer, more transparent, and more sustainable coating systems, increased harmonization of migration limits, and integration of circular-economy criteria into regulatory practice.

The next section expands this perspective by examining the sustainability, recyclability, and circularity of metallic packaging across full life-cycle boundaries.

## 5. Circularity and Sustainability of Metallic Packaging

Metallic packaging materials—primarily aluminum and steel—are commonly regarded as paradigmatic examples of circular materials, owing to their intrinsic recyclability, long-standing industrial use, and well-established collection and recovery infrastructures. This perception is historically justified and has supported the widespread adoption of metals in food and beverage packaging for more than a century, particularly where long shelf life, complete barrier performance, and thermal stability are required [2,25].

However, recent research on circular economy and life-cycle performance indicates that recyclability alone is insufficient to characterize the sustainability of metallic packaging systems. Instead, circularity emerges as a system-level outcome, determined by the interaction between material composition, production routes, product design, waste-management pathways, and regulatory frameworks [92,93]. In this context, high recycling rates do not automatically translate into high material circularity if losses, downcycling, or energy-intensive recovery processes occur along the value chain.

For metallic packaging, this distinction is particularly relevant. Aluminum and steel exhibit markedly different sensitivities to energy sourcing, alloy complexity, and contamination during recycling, while multi-material structures and surface coatings introduce additional constraints that are often overlooked in simplified circularity narratives. Moreover, recent empirical studies demonstrate that real recycling performance depends strongly on the configuration of waste-management systems, including selective collection, sorting efficiency, and post-incineration metal recovery, rather than on material properties alone [94,95].

Accordingly, this section re-examines the circularity and sustainability of metallic packaging by moving beyond material-level assertions and adopting a system-oriented perspective. Life-cycle assessment, recycling efficiency, and environmental trade-offs are discussed in relation to aluminum, steel, stainless steel, and metal-based composites, with explicit consideration of waste-management pathways, technological limits, and policy-driven design-for-recycling requirements.

**Figure 12:**
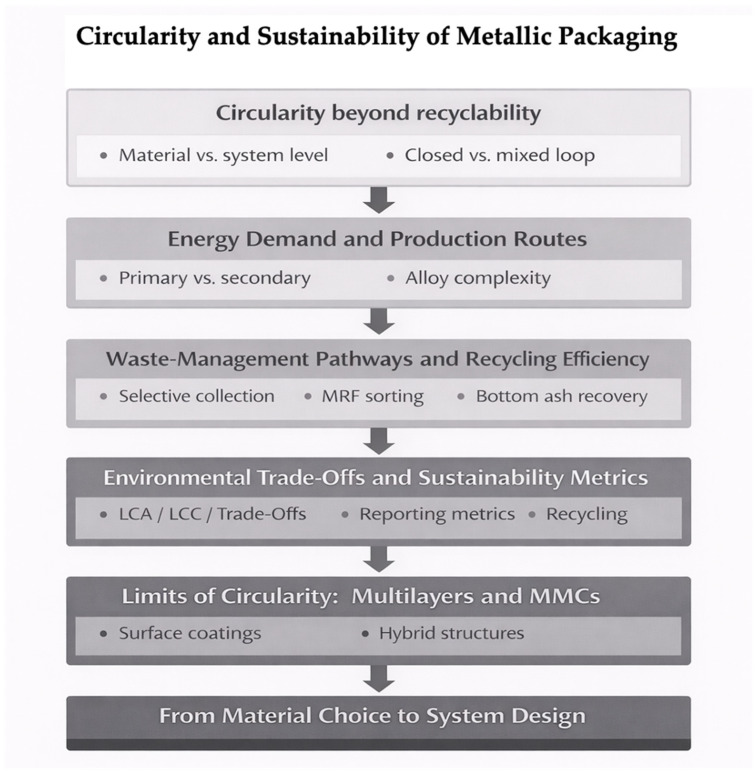
Conceptual roadmap of the section on circularity and sustainability of metallic packaging. The diagram outlines the logical structure of the section, progressing from the conceptual framing of circularity beyond recyclability to energy demand and production routes, waste-management pathways, environmental trade-offs, and the limits of circularity associated with complex packaging architectures, before converging toward a system-design perspective.

Figure 12 provides an overview of the logical structure of this section, highlighting the key dimensions addressed in the analysis of metallic packaging circularity.

### 5.1. Circularity Beyond Recyclability: A Life-Cycle Perspective

In the context of metallic packaging, circularity cannot be equated with recyclability in a strict material sense. While aluminum and steel are theoretically recyclable without intrinsic degradation of their metallic lattice, life-cycle studies demonstrate that effective circularity depends on how materials circulate within real industrial and waste-management systems [96]. This distinction is critical for packaging applications, where high material turnover, short service life, and heterogeneous waste streams amplify the gap between theoretical recyclability and actual material retention.

From a life-cycle perspective, circularity emerges from the capacity of a system to retain material value across successive use cycles while minimizing energy demand, emissions, and losses. In metallic packaging, this capacity is influenced by several interdependent factors, including the structure of recycling loops, the purity of recovered streams, and the compatibility of secondary materials with primary-grade specifications. Studies adopting multi-loop LCA frameworks show that closed product loops—such as can-to-can recycling for aluminum—yield substantially higher environmental benefits than open or mixed recycling loops, where downcycling and dilution with primary material are often unavoidable [96].

Recent meta-analyses of packaging LCAs further confirm that circular performance cannot be captured by single indicators such as recycling rate or recycled content alone. Instead, results are highly sensitive to system boundaries, allocation methods, and end-of-life modelling assumptions, particularly with respect to recycling credits and avoided burdens [93]. As a consequence, two packaging systems with similar reported recycling rates may exhibit markedly different environmental profiles when assessed over their full life cycle.

For metallic packaging, these methodological insights have direct practical implications. Aluminum systems are particularly sensitive to the structure of recycling loops and to alloy management, as impurity accumulation can progressively reduce the substitutability of secondary material for primary aluminum in high-performance applications. Steel systems, while generally more tolerant to compositional variability, remain dependent on effective separation and controlled impurity levels to maintain material quality across cycles. In both cases, circularity is ultimately constrained not by recyclability in principle, but by the alignment between product design, metallurgical requirements, and the operational characteristics of waste-management infrastructures.

This system-oriented interpretation of circularity provides the conceptual basis for the following sections. Energy demand and production routes are examined first, as they dominate the environmental profile of metallic packaging (Section 5.2), followed by an analysis of real recycling pathways and recovery efficiencies within contemporary waste-management systems (Section 5.3).

### 5.2. Energy Demand, Alloy Complexity and Production Routes

Energy demand remains the dominant contributor to the environmental footprint of metallic packaging across most life-cycle assessments. The contrast between primary and secondary production routes is particularly pronounced for aluminum, whose primary production relies on the energy-intensive Hall–Héroult electrolysis process. Multiple life-cycle studies consistently indicate that secondary aluminum production from post-consumer scrap requires only a small fraction of the energy needed for primary aluminum, provided that scrap streams are sufficiently clean and efficiently processed. Critical reviews of aluminum packaging recycling report energy savings on the order of 90–95% for secondary routes relative to primary production, underscoring the central role of recycling in mitigating the environmental burden of aluminum packaging systems [97].

However, the environmental advantage of secondary aluminum is not uniform across all recycling configurations. Alloy complexity and impurity accumulation progressively constrain the extent to which recycled aluminum can substitute primary material in demanding packaging applications. Multi-loop life-cycle analyses show that closed product loops, such as can-to-can recycling, preserve alloy composition and enable repeated substitution of primary aluminum with minimal performance penalties, whereas mixed scrap loops often require dilution with virgin material to meet compositional specifications [96]. As a result, the effective circularity of aluminum packaging depends not only on recycling rates but also on the structure and selectivity of recycling loops.

Steel packaging exhibits a different balance between primary and secondary production. Although the energy gap between blast-furnace routes and electric arc furnace (EAF) steelmaking is smaller than for aluminum, steel benefits from a high tolerance to compositional variability and from mature metallurgical processes that accommodate high scrap fractions without significant degradation of mechanical properties. Life-cycle assessments, therefore, tend to show more stable environmental performance for steel across different recycling scenarios, albeit with lower sensitivity to loop closure compared to aluminum [2]. Nevertheless, steel production remains influenced by scrap availability and by the energy mix supporting EAF operations, linking its circular performance to broader industrial and infrastructural factors.

Beyond bulk production routes, recent studies highlight the relevance of downstream processing steps, such as rolling and forming, in shaping the life-cycle impacts of metallic packaging. For aluminum in particular, hot and cold rolling stages contribute non-negligibly to cumulative energy demand, especially when primary aluminum is involved [98]. These findings reinforce the view that circularity gains achieved through recycling can be partially offset if secondary material is repeatedly reprocessed under energy-intensive conditions, emphasizing the need for system-wide optimization rather than isolated material substitutions.

Overall, energy demand and production pathways define the upper bound of achievable sustainability for metallic packaging. While high recycling rates are a necessary condition for reducing environmental impacts, their effectiveness is mediated by alloy management, loop structure, and process efficiency. These constraints motivate a closer examination of real recycling pathways and recovery efficiencies, which determine how much of the theoretical circular potential of metals is realized in practice, as discussed in the following section.

### 5.3. Recycling Efficiency, Purity of Recovered Streams and Real Recovery

The effective circularity of metallic packaging is ultimately determined by how materials are recovered within real waste-management systems. While life-cycle assessments often assume idealized recycling scenarios, empirical studies demonstrate that recycling yields, material quality, and energy demand vary substantially depending on collection schemes, sorting efficiency, and post-treatment routes. For metallic packaging, this distinction is particularly relevant, as recovery pathways range from selective collection and material recovery facilities to post-incineration treatment of bottom ash.

Selective collection systems generally offer the highest potential for closed-loop recycling, especially for aluminum beverage cans and steel packaging with dedicated collection streams. However, even under optimized collection, material losses occur during sorting, shredding, and remelting. Comparative analyses of European waste-management systems indicate that reported recycling rates based on collected material may systematically overestimate effective material recovery when process losses and downgraded fractions are not fully accounted for [94]. This discrepancy highlights the need to distinguish between nominal recycling performance and actual circular retention of metallic material.

Material recovery facilities (MRFs) play a central role in mixed-waste scenarios, where metals are separated from heterogeneous streams. Steel packaging benefits from intrinsic magnetic properties, enabling robust separation with high capture efficiency across a wide range of waste compositions. Aluminum recovery, by contrast, relies on eddy-current separation and is more sensitive to particle size, shape, and contamination. Empirical assessments show that aluminum recovery efficiency in MRFs decreases markedly for thin foils, laminated structures, and small packaging formats, which are increasingly prevalent in flexible packaging applications [95].

Post-incineration recovery from bottom ash represents an additional, and often underestimated, pathway for metallic packaging circularity. Several studies focusing on European incineration systems demonstrate that significant fractions of aluminum and steel can be recovered from bottom ash using advanced dry and wet treatment processes. Warrings and Fellner (2021) [99] report that, under optimized conditions, aluminum recovery from bottom ash can contribute substantially to overall recycling rates, although with increased energy demand and partial oxidation losses. Importantly, bottom-ash recovery alters the interpretation of recycling statistics, as material recovered after incineration may be counted differently depending on regulatory definitions and reporting boundaries.

Recent reviews emphasize that the contribution of bottom-ash recovery is particularly relevant for aluminum, where thin and lightweight packaging formats are more likely to bypass selective collection and enter residual waste streams [95]. However, aluminum recovered from bottom ash often exhibits reduced quality due to oxidation and alloy contamination, limiting its suitability for high-grade packaging applications and reinforcing the need for dilution with primary material or downcycling into less demanding uses.

These findings underscore that real recycling efficiency is a system-dependent outcome rather than a fixed material property. High recycling rates can be achieved through different pathways, but their implications for energy use, material quality, and effective circularity differ substantially. From a circular-economy perspective, selective collection and closed-loop recycling remain the most favourable routes for metallic packaging, while reliance on mixed-waste processing and bottom-ash recovery introduces additional trade-offs that must be explicitly considered in sustainability assessments.

The limitations observed at the waste-management level become even more pronounced for complex packaging architectures, such as multilayer laminates and coated systems, where recovery efficiency is constrained by technological and economic factors. These aspects are addressed in the following section, which focuses on material-level barriers to circularity.

### 5.4. Environmental Trade-Offs and Sustainability Metrics

The sustainability performance of metallic packaging cannot be reduced to a single environmental indicator. Comparative assessments consistently show that improvements in one dimension—such as energy savings through lightweighting or recycling—may introduce penalties in others, including material losses, additional processing steps, or increased system complexity. As a result, the environmental profile of aluminum- and steel-based packaging is best interpreted through a multi-criteria perspective that explicitly accounts for trade-offs across the life cycle.

Life-cycle costing (LCC) and externality-based analyses comparing aluminum and tinplate packaging illustrate this point clearly. Albuquerque et al. (2019) [50] demonstrated that aluminum packaging benefits from reduced transport emissions and lower use-phase impacts due to lightweighting, whereas tinplate exhibits more stable performance across production and recycling stages, with reduced sensitivity to alloy composition and contamination. Depending on the weighting of climate-related impacts, resource use, or economic costs, either material may appear preferable, underscoring the context-dependent nature of sustainability rankings.

Meta-analyses of packaging LCAs further confirm that divergent conclusions across studies are often driven by methodological choices rather than by fundamental material differences. Bher and Auras (2024) [93] showed that system boundaries, allocation methods for recycling credits, and assumptions regarding end-of-life treatment exert a dominant influence on calculated impacts. In particular, the use of avoided-burden approaches can amplify the apparent benefits of recycling, while cut-off or substitution-based methods may yield more conservative estimates. These methodological sensitivities reinforce the need for transparent and harmonized assessment frameworks when comparing metallic packaging systems.

Within this broader context, recent critical reviews of aluminum packaging recycling highlight an important internal trade-off. While secondary aluminum production offers substantial reductions in energy demand and greenhouse gas emissions relative to primary routes, additional processing steps required for real packaging waste streams—such as decoating, delamination, and melt refining—introduce non-negligible energy and material penalties [97]. These penalties become particularly relevant for complex packaging formats and mixed scrap streams, where the theoretical benefits of recycling may be partially offset by increased processing intensity or by the need for dilution with primary aluminum to maintain alloy specifications.

The trade-off perspective is especially relevant when interpreting comparative sustainability visualizations, such as radar charts or multi-criteria matrices. As illustrated by the radar analysis presented in this work, aluminum typically excels in packaging weight and recyclability, while being penalized by the embodied energy of primary production. Steel, by contrast, exhibits more balanced performance across categories, benefiting from robust recycling pathways and lower sensitivity to alloy purity, but incurring higher transport-related impacts due to density. Metal–matrix composites, although functionally advantageous in niche applications, remain environmentally unfavourable under most assessment frameworks because of high embodied energy and limited recyclability.

Overall, environmental trade-offs in metallic packaging are not anomalies but structural features of complex material systems. Their explicit recognition is essential to avoid oversimplified sustainability narratives and to support informed material and design choices. In this perspective, the value of sustainability metrics lies not in identifying a single “best” material, but in clarifying how design priorities, recycling pathways, and assessment assumptions interact to shape environmental outcomes.

### 5.5. Limits of Circularity: Multilayers and Metal–Matrix Composites

Despite the favourable recyclability of metallic substrates, certain packaging architectures introduce intrinsic limits to circularity that cannot be resolved through recycling optimization alone. Multilayer structures and metal–matrix composites exemplify cases in which functional performance is achieved at the expense of material separability, stream purity, and effective loop closure.

Aluminum–polymer laminates are widely used in flexible and specialty packaging to combine barrier performance, mechanical integrity, and product protection. However, the intimate bonding between metallic foils and polymeric layers complicates end-of-life treatment. While several technological solutions for delamination and material separation have been proposed—including thermal, chemical, and solvent-based routes—their applicability remains constrained by scale, energy demand, and economic viability. Critical analyses of aluminum packaging recycling emphasize that, in most current systems, laminated structures are incompatible with true closed-loop recycling and are more likely to be downcycled or recovered with reduced material quality [97,98,100]. Consequently, the circular performance of such systems is strongly dependent on design choices made upstream, particularly with respect to layer thickness, adhesive chemistry, and compatibility with existing recycling infrastructures.

Similar, but more pronounced, limitations apply to metal–matrix composites. In these systems, ceramic reinforcements such as silicon carbide or oxide particles are intentionally introduced to enhance stiffness, thermal stability, or wear resistance. While these properties are advantageous in technical and electronic packaging applications, they fundamentally disrupt conventional metallurgical recycling routes. Experimental and industrial studies confirm that MMCs cannot be efficiently remelted without segregation issues, excessive slag formation, or irreversible contamination of secondary metal streams [101]. As a result, recycling options for MMCs are typically restricted to niche recovery pathways, partial downcycling, or reuse within tightly controlled industrial loops.

From a circular-economy perspective, these material classes highlight the limits of a purely material-centric sustainability narrative. While metals such as aluminum and steel can sustain multiple life cycles under favourable conditions, the introduction of multi-material architectures or permanent reinforcements shifts the balance toward functionality-driven design, where circularity becomes a secondary constraint. In these cases, sustainability must be evaluated in terms of system-level trade-offs, weighing extended functionality or product protection against reduced end-of-life recovery.

These limitations do not imply that multilayers or MMCs should be excluded from packaging applications, but rather that their use requires explicit justification and transparent accounting of end-of-life implications. For metallic packaging systems aiming at high circularity, design-for-recycling principles remain most effective when applied at the earliest stages of material and product development, before structural incompatibilities are locked into the system.

### 5.6. Outlook: From Material Choice to System Design

The analysis presented in this section highlights that the sustainability of metallic packaging cannot be ensured through material selection alone. While aluminum, steel, and stainless steel retain clear advantages in terms of recyclability and functional performance, their effective circularity depends on the coherence between material design, production routes, waste-management pathways, and assessment methodologies. In this perspective, circularity emerges not as an intrinsic material property, but as the outcome of a coordinated system in which technical, infrastructural, and regulatory elements interact.

For high-volume packaging applications, aluminum is expected to maintain a central role in circular strategies, provided that closed-loop recycling is preserved and alloy management remains compatible with repeated can-to-can or sheet-to-sheet cycles. Steel packaging benefits from robust and tolerant recycling infrastructures, making it particularly suited to stable circular systems where material purity can be maintained despite heterogeneous waste streams. Stainless steel, although less common in single-use packaging, offers strong potential for reusable and long-lifetime applications, where extended service life compensates for higher embodied energy.

At the same time, the increasing diffusion of multilayer structures and functionally optimized composites underscores the tension between performance-driven design and circular-economy objectives. As demonstrated throughout this section, such architectures often exceed the practical limits of current recycling systems, shifting the sustainability balance toward functionality rather than material retention. Addressing this tension requires moving beyond incremental recycling improvements and embracing design-for-recycling principles that are explicitly aligned with real waste-management capabilities.

Future progress in metallic packaging sustainability is therefore expected to rely on system-level optimization rather than on isolated material innovations. This includes the development of recycling-compatible coatings, the rationalization of alloy compositions, improved traceability of material flows, and closer integration between packaging design and end-of-life infrastructure. Regulatory initiatives, such as the evolving European framework on packaging waste and circularity, are likely to accelerate this transition by shifting attention from nominal recyclability to demonstrable circular performance.

Ultimately, the circularity of metallic packaging will be determined by the capacity of the entire system to retain material value across multiple life cycles under realistic industrial conditions. Within this framework, metals remain among the most promising packaging materials, not because they are recyclable in principle, but because they can be integrated into circular systems when design, processing, and recovery are treated as interdependent elements rather than independent variables.

## 6. Discussion and Future Perspectives on Metallic Packaging Systems

The analysis presented in Section 3, Section 4 and Section 5 highlights that the performance of metallic packaging systems cannot be interpreted solely in terms of intrinsic material properties, but must be understood as the outcome of complex interactions among metallurgical architecture, surface engineering, processing conditions, regulatory constraints, and end-of-life scenarios. This section provides a critical discussion of the main cross-cutting challenges emerging from the literature and outlines future perspectives for the evolution of metal-based packaging systems.

### 6.1. Safety, Migration, and Coating Integrity

Although metals exhibit excellent intrinsic barrier properties against gases, moisture, and light, their functional performance in food-contact applications is largely governed by the integrity and long-term stability of internal coatings. Evidence accumulated over several decades indicates that even minor coating defects, incomplete coverage, or localized degradation can initiate corrosion processes and metal migration, particularly under acidic or chloride-rich conditions. Early observations already reported measurable increases in aluminum concentration in canned beverages during extended storage, linking migration phenomena to product chemistry, storage time, and acidity [29]. These findings, rather than being obsolete, have proven structurally persistent and remain relevant in contemporary packaging systems, as confirmed by recent investigations on beverage packaging and aluminum cans [102].

Recent studies confirm that coating-mediated degradation often originates from highly localized phenomena rather than from uniform material failure. Wu et al. (2024) [4] demonstrated that pitting corrosion in coated steel cans preferentially initiates at mechanically stressed regions such as side seams, where oxygen ingress, low pH, and coating discontinuities coexist. Similarly, Chang et al. (2024) [29] showed that insufficient degassing or inadequate sealing pressure in hermetically closed containers significantly increases residual oxygen levels, accelerating internal corrosion and reducing shelf life. Taken together, these results highlight a strong coupling between processing parameters, coating integrity, and long-term safety performance [4,29].

Beyond processing variables, growing attention has been directed toward the interaction between mechanical deformation and chemical degradation. Comparable effects have also been reported for aluminum beverage cans, where controlled denting or seam impact increased aluminum migration into acidic products during long-term storage, despite no loss of airtightness [103]. Recent investigations on tinplate systems indicate that coating damage induced during forming, seaming, or closure can act as a trigger for localized corrosion and subsequent metal release, even when nominal coating specifications are met. Pejić et al. (2025) [41] demonstrated that corrosion at the lid–body junction arises from locally damaged organic coatings combined with insufficient tin layer thickness, emphasizing that safety risks may emerge at mechanically strained interfaces rather than from bulk material properties alone.

In this context, increasing evidence suggests that coating failure in metallic packaging is not governed solely by bulk coating properties, but critically by the integrity and adhesion of interfacial layers between the metal substrate, passivation layer, and organic coating. Detailed analyses of tinplate systems have shown that interlayer adhesion loss can precede visible coating damage, acting as a primary mechanism for localized corrosion initiation under mechanical and chemical stress [104]. This interfacial sensitivity is further exacerbated by forming-induced strain and thermal cycling, reinforcing the role of interfaces as critical weak points in real packaging conditions.

Recent developments in coating design increasingly aim to address these interfacial vulnerabilities through multifunctional and nanocomposite architectures. Epoxy-based nanocomposite coatings incorporating inorganic fillers or functional additives have been shown to enhance mechanical robustness, corrosion resistance, and antimicrobial performance simultaneously, thereby improving tolerance to localized damage [49]. Similarly, bio-inspired oil-infused surface treatments have demonstrated reduced electrolyte adhesion and improved corrosion resistance on tinplate substrates, suggesting alternative strategies that mitigate degradation by modifying interfacial wetting and contact phenomena rather than relying solely on barrier thickness [105].

At the same time, regulatory-driven transitions in surface treatments have introduced new challenges. The replacement of hexavalent chromium passivation by chromium-free alternatives has significantly altered the nanoscale chemistry and heterogeneity of tinplate surfaces. Multi-scale characterization of chromium-free passivation layers has revealed pronounced thickness variations and chemical inhomogeneities, leading to spatially variable corrosion responses and reduced coating adhesion in critical regions [43]. These findings indicate that compliance with updated environmental regulations does not automatically translate into equivalent corrosion resistance or long-term safety margins.

Collectively, these studies indicate that safety in metallic packaging cannot be interpreted solely in terms of material selection or coating formulation. Instead, it emerges from a complex interaction among coating integrity, microstructural heterogeneity, mechanical deformation, and process control. From a future perspective, improvements in metallic packaging safety are therefore expected to rely increasingly on tighter control of forming and closure operations, mitigation of localized strain, and integrated assessment of mechanical–chemical degradation pathways, rather than on incremental material modifications alone [41,43].

### 6.2. Toxicological Concerns and Regulatory Gaps

From a toxicological perspective, migration from food-contact coatings remains one of the most sensitive and debated aspects of metallic packaging safety. Early studies demonstrated that epoxy-based linings could release bisphenol A (BPA) into food matrices under certain storage and thermal conditions, even when migration levels complied with regulatory limits. Recent reviews have confirmed that polymeric can coatings represent complex formulations containing residual monomers, additives, and reaction by-products that may migrate into food products and therefore require detailed analytical monitoring [106]. These investigations played a foundational role in shaping subsequent risk assessments and regulatory scrutiny, establishing migration as a coating-driven rather than substrate-driven phenomenon [20,38].

In the past decade, the toxicological focus has progressively shifted from BPA itself to the broader class of bisphenol analogues and non-intentionally added substances (NIAS) associated with alternative coating formulations. Recent reviews indicate that “BPA-free” claims do not necessarily correspond to the absence of endocrine-active or insufficiently characterized migrants, particularly under elevated temperature, long storage times, or aggressive food chemistries. Kajiyama et al. (2025) [107] highlighted that many epoxy substitutes and alternative resin systems remain only partially assessed from a long-term toxicological standpoint, revealing a gap between regulatory compliance and comprehensive exposure evaluation.

This gap has become increasingly evident in the context of recent regulatory developments. The progressive restriction and subsequent ban of bisphenols in food-contact materials within the European Union have accelerated the transition toward new coating chemistries, often adopted under industrial pressure and tight implementation timelines. While these measures represent a decisive step toward improved consumer protection, they also expose limitations in current regulatory frameworks, which are largely substance-specific and may not fully account for complex migration scenarios involving degradation products, reaction by-products, and NIAS formed during processing or ageing.

In addition to substance-specific regulatory gaps, growing attention has been directed toward discrepancies between regulatory thresholds and real-world exposure scenarios. Comprehensive compilations of aluminum concentrations in foods and beverages indicate that cumulative dietary intake may approach or exceed tolerable intake levels in specific consumer groups, even in the absence of regulatory non-compliance at the individual packaging level [15]. This highlights a structural limitation of current frameworks, which typically assess migration and safety on a single-material or single-contact basis, rather than considering aggregate exposure across multiple sources and packaging systems.

Furthermore, recent comparative analyses of international regulatory approaches reveal significant differences in migration limits, testing protocols, and risk interpretation across regions, including the European Union, the United States, and Asian markets. These divergences complicate both industrial implementation and scientific risk assessment, particularly for globally distributed packaging solutions, and underscore the need for greater harmonization between regulatory requirements and evolving scientific evidence [87].

Recent analytical advances further reinforce this concern. Studies employing high-sensitivity detection methods have demonstrated that trace-level metal ions and organic migrants can still be released from coated metal packaging under realistic storage conditions, particularly in acidic or high-salt foods. The development of advanced sensing and monitoring approaches for aluminum and transition-metal ions illustrates the growing emphasis on post-market verification and continuous safety assessment, rather than reliance on initial compliance testing alone [91].

Collectively, these findings indicate that future toxicological risk management in metallic packaging is likely to evolve from a static, formulation-based approach toward a more dynamic framework integrating migration monitoring, ageing effects, and realistic use conditions. Rather than focusing exclusively on the elimination of individual substances, emerging strategies emphasize the need for transparent characterization of coating systems, long-term exposure assessment, and harmonized regulatory tools capable of addressing complex multilayer and composite architectures. In this context, the transition from compliance-driven approval to verification-oriented safety assessment represents a key challenge and opportunity for next-generation metallic packaging systems [91,107].

### 6.3. Circularity Limits of Coated and Hybrid Systems

Aluminum and steel remain among the most efficiently recycled packaging materials, with well-established collection infrastructures and high recovery rates in many regions. Their intrinsic recyclability and the significant energy savings associated with secondary production have long been central arguments in favour of metallic packaging. However, the progressive evolution toward coated, multilayer, and hybrid architectures has introduced new limitations that increasingly challenge this traditional circularity narrative.

A primary source of complexity arises from organic coatings and surface treatments, which are essential for ensuring corrosion resistance and food safety but may interfere with recycling operations. Residual coatings can contaminate recycled metal streams, affect alloy purity, or require additional thermal or chemical treatments for removal, thereby increasing energy demand and environmental burden. Life-cycle assessment studies on tinplate packaging have shown that, while recycling remains beneficial overall, auxiliary materials and processing steps—including coatings, printing layers, and surface treatments—contribute non-negligibly to the overall environmental footprint and introduce trade-offs across different impact categories [51].

These challenges are amplified in hybrid and multilayer systems combining metals with polymers, paper, or functional interlayers. Such architectures are increasingly adopted to enhance barrier performance, mechanical robustness, or sustainability credentials, yet they often rely on strong interfacial bonding that complicates end-of-life separation. Recent work on paper–aluminum laminates illustrates that advanced design and modelling tools can optimize mechanical performance and material efficiency, but also confirms that coupled systems inherently require more complex recycling pathways compared to mono-material solutions [76].

In this context, recent research increasingly emphasizes design-driven strategies aimed at mitigating circularity losses at the earliest stages of packaging development. Rather than focusing exclusively on end-of-life separation technologies, several studies highlight the role of structural optimization, material efficiency, and predictive modelling in reducing the overall environmental burden of metallic packaging systems. Geometric optimization and thickness reduction approaches have demonstrated that significant material savings can be achieved without compromising mechanical integrity or safety requirements, thereby lowering the absolute impact of coatings and auxiliary layers throughout the life cycle [69].

Complementarily, the adoption of digital design tools, such as digital twin models for multilayer packaging architectures, enables the simultaneous evaluation of mechanical performance, barrier function, and recyclability constraints before industrial implementation. Recent applications to paper–aluminum laminates illustrate how performance-driven hybrid designs can be quantitatively assessed against circularity metrics, supporting more informed trade-offs between functionality and end-of-life complexity [76]. When combined with life cycle assessment frameworks, these approaches confirm that circularity in coated and hybrid metallic packaging increasingly depends on integrated design choices rather than on recycling efficiency alone [51].

From a circular-economy perspective, this evolution highlights a fundamental tension between design-for-function and design-for-recycling. Performance-driven solutions—such as multilayer coatings, hybrid laminates, and multifunctional barriers—tend to increase material heterogeneity and interfacial complexity, which may undermine recyclability if not explicitly addressed during the design phase. As emphasized in the recent literature, future metallic packaging systems must therefore integrate recyclability constraints at the earliest stages of material and structural design, rather than treating end-of-life considerations as a downstream problem.

Looking forward, several strategic directions emerge. These include the development of coatings that are either compatible with existing recycling processes or readily removable under mild conditions, the prioritization of mono-material dominance in multilayer designs, and the adoption of disassemblable or weakly bonded interfaces where hybridization is unavoidable. Without such design-for-recycling strategies, the increasing functional sophistication of metallic packaging risks eroding the circular advantages that have historically distinguished metals from alternative packaging materials.

### 6.4. Emerging Coating Technologies and Advanced Systems

Emerging coating technologies are increasingly proposed as a key pathway to address the simultaneous demands of safety, performance, and sustainability in metallic packaging systems. In contrast to conventional barrier coatings, recent research has focused on multifunctional surface layers capable of combining corrosion protection, antimicrobial activity, and enhanced durability under dynamic processing and storage conditions. These developments reflect a broader shift from passive protection toward functionally active and adaptive coating concepts.

Among the most investigated approaches are nanostructured and hybrid coatings designed to improve both corrosion resistance and hygienic performance. Recent studies on tinplate substrates have demonstrated that composite coatings incorporating graphene-derived phases and functional polymers can significantly enhance resistance to corrosion while providing antibacterial properties, particularly under aggressive food-contact conditions [48]. While such systems highlight the potential of nanostructured coatings to extend service life and reduce contamination risks, their long-term stability, scalability, and regulatory acceptance remain open questions.

In parallel, increasing attention has been directed toward self-healing and stimuli-responsive coatings as a means of mitigating defect-driven degradation. Recent reviews indicate that self-healing mechanisms—based on reversible chemical bonds, microencapsulated healing agents, or dynamic polymer networks—may partially restore coating integrity after mechanical damage, thereby limiting localized corrosion and migration phenomena [108,109]. Although these concepts are still largely confined to laboratory-scale demonstrations, they provide a clear indication of future directions aimed at reducing the sensitivity of metallic packaging to unavoidable mechanical strain during forming and closure.

Beyond protective functions, advanced coating systems are also being explored in conjunction with analytical and monitoring technologies. The integration of sensing capabilities, such as fluorometric detection of trace metal ions, illustrates a complementary strategy in which coating performance and migration behaviour could be continuously assessed rather than inferred solely from pre-market testing [91]. Such approaches align with emerging regulatory expectations for post-market surveillance and long-term safety verification.

Despite these promising developments, several barriers currently limit the widespread adoption of advanced coating technologies in metallic packaging. Industrial scalability, cost-effectiveness, compatibility with high-speed manufacturing processes, and uncertainty regarding end-of-life behaviour remain critical challenges. Moreover, increased functional complexity may exacerbate recycling constraints, particularly if novel coating chemistries are not compatible with existing material recovery streams.

A defining characteristic of emerging coating technologies is the increasing convergence of multiple functionalities within a single surface layer. Recent studies on epoxy-based nanocomposite coatings demonstrate that mechanical reinforcement, corrosion resistance, and antimicrobial activity can be simultaneously enhanced through the incorporation of nanoscale fillers and functional additives [49]. Similar multifunctional behaviour has been reported for coatings based on metal and metal-oxide nanoparticles, which offer improved barrier and antimicrobial performance but also introduce new questions regarding particle release, long-term stability, and toxicological assessment [32].

In response to these concerns, alternative design strategies are gaining attention. Bio-inspired and oil-infused surface treatments have been proposed as a means to improve corrosion resistance and reduce food adhesion without relying on persistent or bioaccumulative nanomaterials, thereby potentially lowering toxicological and regulatory risks [105]. These approaches reflect a broader shift from purely performance-driven coating design toward balanced risk–benefit optimization, in which functional gains are evaluated alongside safety, regulatory acceptance, and end-of-life compatibility [110].

From a future perspective, the successful deployment of emerging coating technologies will therefore depend on their ability to deliver demonstrable safety and performance benefits while maintaining regulatory compliance, industrial robustness, and recyclability. Rather than incremental improvements in coating composition alone, progress is likely to arise from integrated design strategies that jointly address coating chemistry, mechanical durability, process compatibility, and end-of-life considerations.

### 6.5. Strategic Outlook

The future of metallic packaging systems will be shaped by the ability to reconcile safety, performance, and environmental sustainability within increasingly stringent regulatory and circular-economy frameworks. As highlighted throughout this review, many of the critical challenges facing metallic packaging do not originate from intrinsic material limitations but from the growing complexity of surface treatments, multilayer architectures, and processing conditions required to meet modern functional demands.

**Figure 13:**
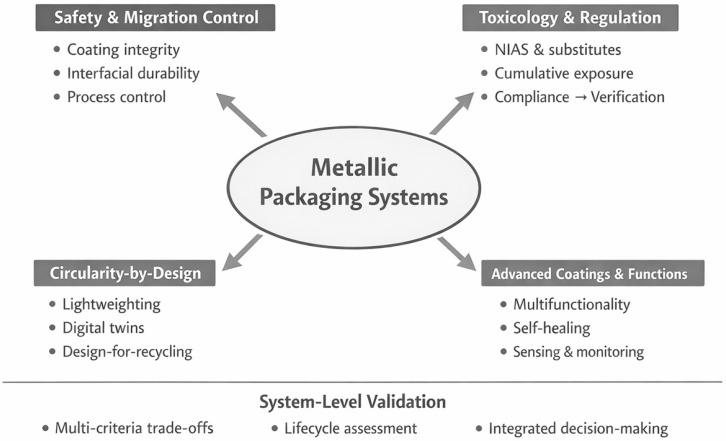
Schematic overview of the main emerging directions shaping future metallic packaging systems. The figure illustrates the convergence of safety and migration control, toxicological and regulatory evolution, circularity-by-design strategies, and advanced coating functionalities toward system-level validation frameworks based on multi-criteria decision-making and life-cycle considerations.

Figure 13 schematically summarizes the main emerging directions and system-level trade-offs shaping future metallic packaging systems, as discussed in Section 6.1, Section 6.2, Section 6.3, Section 6.4 and Section 6.5.

From a safety and toxicological standpoint, future progress is expected to rely less on the substitution of individual substances and more on system-level approaches integrating coating integrity, process control, and long-term verification of migration behaviour. The transition from static compliance-based approval toward verification-oriented safety assessment, supported by advanced analytical and monitoring tools, represents a fundamental shift in how food-contact materials may be regulated and managed over their life cycle [91,107].

In parallel, circularity considerations are likely to become an increasingly decisive design constraint. While metals retain intrinsic advantages in terms of recyclability, the continued expansion of coated, hybrid, and functionally enhanced systems risks eroding these benefits if end-of-life scenarios are not explicitly addressed at the design stage. Life-cycle assessment and digital modelling studies demonstrate that performance-driven solutions must be evaluated against their full environmental footprint, including auxiliary materials, processing steps, and separation requirements [51,76].

Emerging coating technologies and advanced surface systems offer promising pathways to mitigate some of these challenges, particularly by enhancing durability, reducing defect sensitivity, and enabling new safety functions. However, their successful adoption will ultimately depend on industrial scalability, regulatory acceptance, and compatibility with existing recycling infrastructures. In this context, technological innovation alone is insufficient without coordinated advances in manufacturing practices, standardization, and policy alignment.

Overall, the strategic outlook for metallic packaging points toward a shift from material-centric optimization to integrated system design. Future developments will increasingly require the joint consideration of metallurgical architecture, surface engineering, processing control, regulatory compliance, and end-of-life performance. By addressing these dimensions in a unified manner, metallic packaging can maintain its relevance and competitiveness in applications where safety, durability, and sustainability must be simultaneously ensured.

## 7. Conclusions

This review has provided a system-level analysis of metallic packaging materials, integrating material-specific properties, processing routes, regulatory constraints, and sustainability considerations into a unified framework. By moving beyond a purely material-centred perspective, the work highlights how the functional performance of metallic packaging arises from the interaction between metallurgical architecture, surface engineering, joining technologies, and end-of-life pathways.

Aluminum, steel, stainless steel, and advanced metal–matrix composites each occupy distinct functional niches that cannot be ranked along a single performance axis. Lightweight aluminum systems dominate high-volume food and beverage packaging due to their formability and recyclability, while coated steel remains the reference solution for mechanically demanding and thermally processed foods. Stainless steels offer unmatched chemical inertness and durability in high-value, reusable, and pharmaceutical applications, whereas metal–matrix composites extend metallic packaging into advanced technical and electronic domains where thermo-mechanical stability outweighs cost and circularity constraints.

A central conclusion emerging from this analysis is that safety and long-term reliability are increasingly governed by surface and interface integrity rather than by bulk metal properties. Coating performance, processing conditions, headspace control, and defect tolerance play a decisive role in migration phenomena, corrosion resistance, and regulatory compliance. These aspects introduce intrinsic trade-offs between functional performance, safety assurance, and recyclability, particularly in coated and multilayer systems.

From a sustainability perspective, metals retain a strong advantage due to their high recycling rates and established recovery infrastructures. However, the growing complexity of coatings, hybrid architectures, and composite systems poses new challenges for circularity, material purity, and end-of-life management. Addressing these challenges requires a shift toward design-for-recycling strategies, simplified material architectures, and improved compatibility between functional layers and recycling processes.

Overall, the findings of this review underscore that metallic packaging should be conceived as an engineered system rather than as a single-material solution. Future developments will depend on the ability to integrate safety, performance, regulatory compliance, and circularity within coherent system designs. In this context, the continued evolution of metallic packaging will rely not only on incremental material improvements but also on coordinated advances in surface technologies, processing control, and life-cycle-oriented design principles.

## Figures and Tables

**Table 1:** Comparative technical overview of aluminum alloys used in packaging.

Alloy Series	Typical Grades	Yield Strength (MPa) *	Typical Thickness	Strengthening Mechanism	Corrosion Behaviour in Food Environments	Coating Requirement	Main Packaging Applications	Representative References
1xxx	Commercially pure Al	30–120	6–50 μm	Solid solution + strain hardening	Good intrinsic resistance; sensitive to pinholes in thin foil	Often laminated	Foils, lidding	[1,2]
3xxx	AA3004, AA3104	140–280 (H14–H19)	0.20–0.30 mm	Al–Mn solid solution + work hardening	Susceptible to localized pitting in acidic beverages	Mandatory internal lacquer	Beverage can bodies (DWI)	[6,17,37]
5xxx	AA5182	200–320	0.20–0.28 mm	Al–Mg solid solution + work hardening	Moderate corrosion sensitivity; critical at score lines	Mandatory internal lacquer	Can ends, closures	[4,6,36]
8xxx	AA8011	100–200	6–20 μm	Fe–Si intermetallic dispersoids + work hardening	Stable when laminated; failure governed by defects	Laminate-dependent	Blisters, retort laminates	[1,39]

* Strength values depend on temper condition and represent indicative ranges reported in the literature for packaging-relevant tempers.

**Table 2:** Comparative technical overview of electrolytic tinplate systems (ETP).

System Type	Base Steel Grade	Yield Strength (MPa) *	Typical Thickness (mm)	Protective Layer	Corrosion Behaviour	Coating Requirement	Main Packaging Applications	Representative References
Tinplate (ETP)	Low-carbon steel (MR grades)	280–550	0.15–0.35	Electrolytic tin coating (≈1–11 g/m^2^)	Corrosion governed by coating integrity and electrochemical coupling between Sn and Fe	Mandatory internal lacquer	Food cans, aerosol containers	[2,25]
Double-reduced (DR) tinplate	Cold-work strengthened low-carbon steel	400–650	0.14–0.22	Electrolytic tin coating	Higher strength, reduced formability; corrosion still coating-dependent	Mandatory internal lacquer	Shallow-drawn cans, beverage steel cans	[2,25]

* Strength values depend on temper condition (T1–T5, DR grades) and represent indicative ranges reported in the literature for packaging-relevant applications.

**Table 3:** Comparative technical overview of tin-free steel (TFS/ECCS).

System	Base Material	Yield Strength (MPa) *	Typical Thickness (mm)	Protective Layer	Corrosion Behaviour	Coating Requirement	Main Packaging Applications	Representative References
TFS/ECCS	Low-carbon steel	280–550	0.15–0.30	Chromium/chromium oxide duplex layer	Corrosion protection entirely coating-dependent; no sacrificial behaviour	Mandatory internal lacquer	Easy-open ends, lids, closures	[2,25]

* Strength values depend on temper condition and correspond to substrate grades used for packaging applications.

**Table 4:** Comparative technical overview of stainless steels used in packaging.

Stainless Grade	Microstructural Class	Yield Strength (MPa)	Young’s Modulus (GPa)	Typical Thickness (mm)	Corrosion Behaviour in Food Environments	Coating Requirement	Main Packaging Applications	Representative References
AISI 304	Austenitic (Cr–Ni)	200–300	~200	0.2–0.6	Excellent general corrosion resistance; susceptible to chloride-induced pitting	Not mandatory	Food containers, specialty packaging	[2,25]
AISI 316	Austenitic (Cr–Ni–Mo)	200–300	~200	0.2–0.6	Improved pitting resistance due to Mo addition	Not mandatory	Aggressive food and pharma environments	[2]
AISI 430	Ferritic (Cr)	250–350	~200	0.2–0.5	Moderate corrosion resistance; lower cost	Not mandatory	Selected packaging components	[25]

**Table 5:** Advanced metal–matrix composite architectures for electronic and high-power device packaging.

MMC System	Matrix	Reinforcement	Targeted Property/Function	Typical Processing Route	Key Interface/Surface Strategy	Typical Packaging Component(s)	Main Limiting Factor(s)	Representative References
Al–SiC (particle/preform MMC)	Al	SiC particles/SiC preform (high Vf)	CTE tailoring (~7–10 × 10^−6^ K^−1^) for Si/ceramic matching; high stiffness; good thermal stability	Squeeze casting; pressure infiltration; PM routes	Wetting control; porosity suppression; uniform reinforcement distribution	Baseplates, heat spreaders, module substrates	Reduced ductility; process sensitivity at high Vf	[13,61,62]
Al–diamond (bulk MMC)	Al	Diamond particles (surface-treated)	Ultra-high thermal conductivity (>500–600 W·m^−1^·K^−1^) for high heat-flux dissipation; CTE engineering	Low-pressure infiltration; pressure-assisted infiltration	Interfacial engineering to limit brittle/moisture-sensitive phases (e.g., Al_4_C_3_); tailored interlayers/coatings	Heat spreaders, high-power baseplates, optoelectronic packages	Cost; interfacial stability/reaction control	[30,34]
Al–diamond (laser-based MMC layer/local reinforcement)	Al alloy	Diamond (local MMC region)	Local thermal flow and stress tailoring within housing/substrate; integration without full bulk MMC	Laser cladding/laser deposition	Local interface control; designed MMC geometry; mitigation of defects (porosity/weak bonding)	Locally reinforced housings/heat-spreader regions	Property variability vs. bulk infiltration routes; processing constraints	[35]
Cu–SiC (MMC for power modules)	Cu	SiC particles/network	High stiffness and dimensional stability; improved thermo-mechanical fatigue resistance; CTE management vs. ceramics/SiC	Infiltration/PM routes (application-driven)	Metallization (Ni, Ni–P, and Au) for assembly/solder compatibility; interface stability in joined stacks	Power-electronics baseplates, module substrates, housings	Density/cost; joining complexity; galvanic/interface heterogeneity	[12,60,63]
MMC-enabled lightweight baseplates (application-driven design)	Al- or Cu-based MMC	Ceramic/carbon reinforcements (design-specific)	Weight saving while maintaining thermal performance and reliability (system-level optimization)	Design + numerical/thermal optimization; architecture selection (application-led)	Interface and joint design to preserve flatness and fatigue resistance under cycling	Aeronautic electronic packaging baseplates	Manufacturing complexity; cost; qualification burden	[65]

*Reported properties depend on reinforcement fraction, morphology, processing route and interface design; values are therefore representative of packaging-oriented MMC architectures rather than fixed material constants.*

**Table 6:** Technical comparison of metallic materials for packaging. Technical comparison of representative metallic materials used in packaging systems. Indicative ranges refer to packaging-grade materials and depend on alloy composition, temper, processing route, and surface condition.

Property	Aluminum [4]	Coated Steel [82]	Stainless Steel [38]	Al–Diamond Composite [30]
**Composition**	Aluminum packaging alloys (typically Al–Mn or Al–Mg systems)	Low-carbon steel substrate with electrolytic tin (tinplate) or chromium/chromium oxide coating (ECCS)	Steel alloy with Cr ≥ 10.5% and possible Ni	Aluminum matrix reinforced with diamond or SiC particles (typically 40–70 vol.%)
**Characteristics**	Lightweight, recyclable, intrinsic metallic barrier	High strength, cost-effective, coating-dependent performance	Chemically stable, reusable, passive surface	High thermal conductivity, low thermal expansion
**Corrosion resistance**	Moderate, coating-enhanced	Low without coating; coating-dependent	Very high (passive surface)	Moderate, interface-dependent
**Thermal conductivity (W/m·K)**	205	50	16–25	>200
**Young’s modulus (GPa)**	70–80	190–210	190–210	150–300
**Tensile strength (MPa)**	100–300	350–600	500–900	200–300
**Density (g/cm^3^)**	2.7	7.85	7.9–8.1	2.8
**Poisson’s ratio**	0.33	0.30	0.27–0.30	0.25
**Relative hardness**	Low	Medium	High	Medium–High
**Fracture toughness (MPa·m^1/2^)**	20–30	50–70	80–120	15–25
**Barrier properties (O_2_) (cc/m^2^·24 h)**	0.01–0.02	0.1–0.3 (with coating)	Negligible (passive surface)	0.1–0.2
**Barrier properties (H_2_O) (g/m^2^·24 h)**	0.1–0.2	High, coating-dependent	*Near-zero*	0.1–0.3
**Barrier (Light/UV)**	*Intrinsic metallic opacity*	*Intrinsic metallic* *opacity*	*Intrinsic metallic opacity*	*Intrinsic metallic* *opacity*
**Major applications**	Food, pharmaceutical, and cosmetics	Food cans, caps, and closures	Reusable bottles, medical, and premium food	Electronics, aerospace

**Table 7:** Regulatory and standardization framework for metallic packaging systems.

Function	EU	US	ISO/International	Application Sectors
Legal framework	Regulation (EC) 1935/2004	FDA 21 CFR	—	Food; Pharma; Reusable **
Migration limits	EFSA SML/TDI	FDA limits	—	Food; Pharma *
Test methods	EN 1186	FDA methods	ISO 9227	Food; Reusable; Pharma *
Application-specific requirements	—	USP <661>, <671>	—	Pharma

* Applied as baseline or by analogy; ** general safety principles extended to reusable systems.

## Data Availability

No new data were created or analyzed in this study. Data sharing is not applicable to this article.

## Appendix A. Regulatory and Standardization Framework for Metallic Packaging

This appendix summarizes the main regulatory instruments and technical standards referenced in Section 4, which govern the safety, migration behaviour, corrosion resistance, and functional performance of metallic packaging systems. These documents define mandatory requirements, test methodologies, and compliance criteria applicable to food-contact, pharmaceutical, reusable, and technical metallic packaging.

### Appendix A.1. European Union Regulatory Framework

- Regulation (EC) No 1935/2004

Framework regulation on materials and articles intended to come into contact with food, establishing the general safety requirement that such materials must not endanger human health, alter food composition, or affect organoleptic properties.

- Commission Regulation (EU) No 10/2011

Regulation on plastic materials and articles intended to come into contact with food; applied in metallic packaging primarily to polymeric coatings and lacquers used on metal substrates.

- EN 1186

Materials and articles in contact with food—Test methods for overall and specific migration into food simulants. European Committee for Standardization (CEN): Brussels, Belgium, 2013.

- EN 13130

Materials and articles in contact with food—Test methods for specific migration from active and intelligent materials. European Committee for Standardization (CEN): Brussels, Belgium, 2013.

- EN 602:2004

Tinplate—Technical delivery conditions. European Committee for Standardization (CEN): Brussels, Belgium, 2004.

- EN 10333

Steel flat products for packaging—Tinplate and electrolytic chromium-coated steel (TFS/ECCS). European Committee for Standardization (CEN): Brussels, Belgium, 2005.

- EFSA guidance documents

Scientific opinions and guidelines defining tolerable intake values and specific migration limits (SMLs) for metals such as aluminum, tin, chromium, nickel, and alloying elements used in food-contact materials.

- Council of Europe technical guidelines on metals and alloys in contact with food

Technical reference documents defining specific release limits (SRLs), simulants, and test conditions for compliance assessment of metallic food-contact materials.

### Appendix A.2. International and ISO Standards

- ISO 9227:2023 (UNI EN ISO 9227:2023)

Corrosion tests in artificial atmospheres (salt spray), widely used for comparative corrosion benchmarking of coated metallic packaging. International Organization for Standardization: Geneva, Switzerland, 2023.

- ISO 4624

Paints and varnishes—Pull-off test for assessment of coating adhesion strength on metallic substrates. International Organization for Standardization: Geneva, Switzerland, 2016.

- ISO 8301

Packaging—Determination of lacquer continuity and defect detection on coated metal surfaces. International Organization for Standardization: Geneva, Switzerland.

- ISO 4531

Vitreous and porcelain enamels—Release of lead and cadmium; in some jurisdictions, test principles are extended by analogy to assess migration from metallic or coated systems. International Organization for Standardization: Geneva, Switzerland.

### Appendix A.3. United States Regulatory and Pharmacopeial Standards

- FDA Title 21 Code of Federal Regulations (21 CFR)

U.S. regulatory framework defining permitted materials, substances, and conditions of use for food-contact materials, including metallic substrates and organic coatings.

- NSF standards

Standards applicable to reusable metallic containers and food-contact equipment, addressing hygiene, cleanability, and material safety.

- United States Pharmacopeia (USP)
  ○ USP <661>—Plastic packaging systems and materials of construction (applied by analogy to coated metallic pharmaceutical containers).
  ○ USP <671>—Containers—Performance testing, including permeation and integrity requirements for pharmaceutical packaging.

### Appendix A.4. Scope and Use Within This Review

The standards and regulations listed above are cited in the main text to clarify:

- Applicable legal and regulatory constraints;
- Standardized methods used for migration, corrosion, and coating-performance assessment;
- Differences between food, pharmaceutical, reusable, and technical packaging requirements.

They are referenced as normative documents rather than scientific literature and are therefore reported in this appendix instead of the main reference list.
